# Tissue Mechanics and Hedgehog Signaling Crosstalk as a Key Epithelial–Stromal Interplay in Cancer Development

**DOI:** 10.1002/advs.202400063

**Published:** 2024-07-08

**Authors:** Shanika Karunasagara, Ali Taghizadeh, Sang‐Hyun Kim, So Jung Kim, Yong‐Jae Kim, Mohsen Taghizadeh, Moon‐Young Kim, Kyu‐Young Oh, Jung‐Hwan Lee, Hye Sung Kim, Jeongeun Hyun, Hae‐Won Kim

**Affiliations:** ^1^ Institute of Tissue Regeneration Engineering (ITREN) Dankook University Cheonan 31116 Republic of Korea; ^2^ Department of Nanobiomedical Science & BK21 Global Research Center for Regenerative Medicine Dankook University Cheonan 31116 Republic of Korea; ^3^ Department of Chemistry College of Science & Technology Dankook University Cheonan 31116 Republic of Korea; ^4^ Department of Oral and Maxillofacial Surgery College of Dentistry Dankook University Cheonan 31116 Republic of Korea; ^5^ Department of Oral Pathology College of Dentistry Dankook University Cheonan 31116 Republic of Korea; ^6^ Mechanobiology Dental Medicine Research Center Dankook University Cheonan 31116 Republic of Korea; ^7^ UCL Eastman‐Korea Dental Medicine Innovation Centre Dankook University Cheonan 31116 Republic of Korea; ^8^ Department of Biomaterials Science College of Dentistry Dankook University Cheonan 31116 Republic of Korea; ^9^ Department of Regenerative Dental Medicine College of Dentistry Dankook University Cheonan 31116 Republic of Korea; ^10^ Cell & Matter Institute Dankook University Cheonan 31116 Republic of Korea

**Keywords:** cancer development, chemomechanical cues, epithelial–stroma interplay, hedgehog, tissue stiffness

## Abstract

Epithelial‐stromal interplay through chemomechanical cues from cells and matrix propels cancer progression. Elevated tissue stiffness in potentially malignant tissues suggests a link between matrix stiffness and enhanced tumor growth. In this study, employing chronic oral/esophageal injury and cancer models, it is demonstrated that epithelial–stromal interplay through matrix stiffness and Hedgehog (Hh) signaling is key in compounding cancer development. Epithelial cells actively interact with fibroblasts, exchanging mechanoresponsive signals during the precancerous stage. Specifically, epithelial cells release Sonic Hh, activating fibroblasts to produce matrix proteins and remodeling enzymes, resulting in tissue stiffening. Subsequently, basal epithelial cells adjacent to the stiffened tissue become proliferative and undergo epithelial‐to‐mesenchymal transition, acquiring migratory and invasive properties, thereby promoting invasive tumor growth. Notably, transcriptomic programs of oncogenic GLI2, mechano‐activated by actin cytoskeletal tension, govern this process, elucidating the crucial role of non‐canonical GLI2 activation in orchestrating the proliferation and mesenchymal transition of epithelial cells. Furthermore, pharmacological intervention targeting tissue stiffening proves highly effective in slowing cancer progression. These findings underscore the impact of epithelial‐stromal interplay through chemo‐mechanical (Hh‐stiffness) signaling in cancer development, and suggest that targeting tissue stiffness holds promise as a strategy to disrupt chemo‐mechanical feedback, enabling effective cancer treatment.

## Graphical abstract

This study uncovers a crucial interplay between epithelial and stromal cells in oral/esophageal cancer development, driven by Hedgehog signaling and a stiffened matrix. Sonic Hedgehog‐dependent signaling increases matrix stiffness, facilitating GLI2 translocation into the nucleus through actomyosin‐mediated cytoskeleton tension in adjacent epithelial cells. This mechanism governs oncogenic transcriptomic programs essential for invasive tumor growth during precancerous stages.

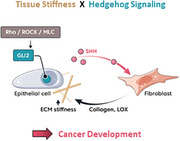

## 1 Introduction

A crosstalk between cancer‐associated fibroblasts (CAFs) and cancer cells plays a pivotal role in driving cancer progression.^[^ ^1^ ^]^ These cells engage in a reciprocal exchange of secretomes, which are signaling molecules supporting their expansion and tumor‐promoting capabilities.^[^ ^2^ ^]^ Chronic injury prompts the activation of fibroblasts, mainly the tissue‐resident fibroblasts. The transformation of these fibroblasts into CAFs is orchestrated by various secreted factors from cancer cells, immune cells, and stromal cells within the tumor microenvironment. Furthermore, a subpopulation of CAFs produces extracellular matrix (ECM) proteins and ECM remodeling enzymes, establishing a desmoplastic microenvironment favorable for malignant transformation and tumor growth.^[^ ^2^, ^3^ ^]^ Indeed, many cancers, including hepatocellular, pancreatic, gastric, esophageal, and head and neck cancers, arise in fibrotic tissues,^[^ ^4^ ^]^ where the collagen‐rich matrices exhibit a pro‐tumorigenic phenotype.^[^ ^5^ ^]^ Of note, not only the amount but also the architecture of collagen fibers plays a decisive role in the mechanical properties of the ECM, particularly its stiffness.^[^ ^6^ ^]^ Lysyl oxidase (LOX) family members derived from CAFs and cancer cells are key enzymes that catalyze collagen crosslinking and the elongation and alignment of collagen fibers, leading to ECM stiffening.^[^ ^7^ ^]^ In turn, cells sense and respond to the stiffness of ECM by altering their morphology, proliferation, migration, and functions.^[^ ^8^ ^]^ A recent study found that mammary epithelial cells lose their epithelial morphology and undergo epithelial‐to‐mesenchymal transition (EMT)‐like process on stiff hydrogel substrates through Transforming growth factor‐β (TGF‐β) and Yes‐associated protein (YAP) signaling, compared to cells in contact with soft substrates.^[^ ^9^ ^]^ Additionally, cancer cells leverage the traction force on the remodeled ECM to increase their migration speed^[^ ^10^ ^]^ while inducing a tumorigenic phenotype through epigenetic reprogramming.^[^ ^11^ ^]^ Therefore, targeting ECM stiffness emerges as a promising avenue for the treatment of solid cancers.

Hedgehog (Hh) signaling plays an important role in various carcinomas, including lung squamous cell carcinoma,^[^ ^12^ ^]^ basal cell carcinoma,^[^ ^13^ ^]^ and oral squamous cell carcinoma (OSCC).^[^ ^14^ ^]^ Canonical activation of Hh signaling involves the binding of Hh ligands to the receptor patched (Ptch), which allows smoothened (Smo) to localize to the tip of primary cilia (PC), where Smo promotes the activation of GLI‐Krϋppel family of zinc‐finger containing transcription factors (GLI‐1, GLI‐2, GLI‐3).^[^ ^15^ ^]^ GLIs are well‐known to exert oncogenic effects by activating genes that contribute to proliferation, cell survival, EMT, genetic instability, cancer stem cell self‐renewal, and angiogenesis.^[^ ^16^ ^]^ A growing body of evidence suggests the interplay between Hh and mechanotransducive effector YAP signaling, where YAP drives *SHH* gene transcription and secreted SHH induces YAP activation in a positive feedback loop. This chemomechanical interaction coordinates tissue growth and patterning, cell specification and differentiation, and metabolic reprogramming.^[^ ^17^ ^]^ For instance, a gradient of mechanical stress and tissue stiffness during morphogenesis leads to regional activation of YAP, and subsequent FoxA2 and SHH‐GLI activation to direct notochord formation and neural tube patterning.^[^ ^17c^ ^]^ As such, investigations of integrated biochemical and biophysical signaling will help to understand the mechanisms of cancer development, progression, metastasis, and different responses to cancer treatment, ultimately guiding effective therapeutic strategies.

While the significance of epithelial‐stromal interplay mediated by chemomechanical cues from cells and ECM in cancer development is acknowledged, the precise mechanisms orchestrating the coordination between matrix stiffness and oncogenic biochemical signals to enhance tumor growth remain elusive. To address this, here we employ oral/esophageal SCC and mouse models of chronic oral/esophageal injury as robust systems for investigating tissue mechanics and intercellular crosstalk.

Recent reports have shown the increased stiffness in potentially malignant (i.e., oral leukoplakia) and OSCC tissues compared to normal oral mucosal tissues. Elevated LOX expression in tumor stroma and increased collagen contents serve as prognostic indicators of poor survival in OSCC patients.^[^ ^18^ ^]^ Analyzing 260 human OSCC tissues, we observe upregulated GLI2 expression alongside an increase in collagen accumulation. Mechanistically, we demonstrate that Hh ligands produced by injured epithelial cells activate fibroblasts into CAF‐like myofibroblasts, leading to increased tissue stiffness. Concurrently, epithelial cells proliferate and undergo EMT to acquire migratory and invasive capabilities through non‐canonical GLI2 activation in response to the stiffened ECM cue. Notably, we establish, for the first time, that tissue stiffness‐induced GLI2 activation is dependent on RhoA/Rho‐associated kinase (ROCK)/Myosin light chain (MLC)‐mediated actin cytoskeletal tension.

Utilizing single nucleus/spatial transcriptomics analyses of human leukoplakia and normal oral mucosal tissues, we further unveil the communication between epithelial cells and fibroblasts through chemo‐mechanical signals, such as collagen and TGF‐β signaling, during precancerous conditions. GLI2‐expressing epithelial cells exhibit specific gene expression patterns and alterations in molecular signatures associated with cancer. Additionally, we demonstrate that inhibiting collagen crosslinking and softening the ECM impede oral/esophageal tumorigenesis by reducing mechanical cues, disrupting the feedback loop of epithelial‐stromal interplay. Our results propose a critical event in oral/esophageal tumorigenesis, revealing a novel mechanism for the activation of oncogenic transcription factor GLI2 in premalignant epithelial cells by tissue stiffness.

## 2 Results

### 2.1 Biochemical Hedgehog Signaling Remodels the Stroma and Increases Matrix Stiffness during Chronic Injury in Tongue and Esophagus

**Figure 1:**
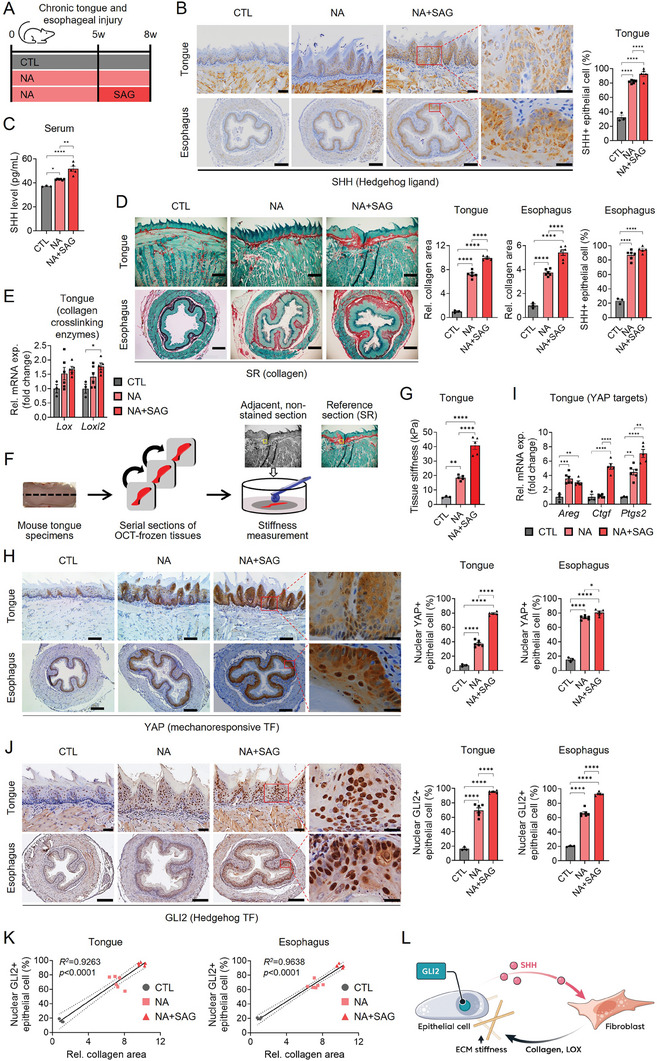
Hedgehog (Hh) signaling increases matrix stiffness which, in turn, activates epithelial YAP and GLI2 during chronic tongue/esophageal injury. A) The in vivo study design and timeline consisted of 8 weeks of exposure to 4‐NQO and arecoline (NA) in drinking water with (*n* = 5) or without (*n* = 6) 3 weeks of additional treatment with Smoothened agonist (SAG, 25 mg kg^−1^ day^−1^, twice a week, *n* = 5) via oral gavage. Age and sex‐matched mice (*n* = 3) that consumed plain water throughout the experimental period were used as the control (“CTL”) group. B) Immunohistochemistry (IHC) for Sonic Hedgehog (SHH) in the tongue and esophagus of CTL, NA, and NA+SAG‐treated mice. The average percentages of SHH‐expressing (SHH+) epithelial cells were quantified. Scale bars = 50 µm (tongue), 200 µm (esophagus), 20 µm (inset). (C) Serum levels of SHH in the mice. D) Sirius red (SR) staining and quantification of the relative collagen area in the tongue and esophageal tissue sections of CTL, NA, and NA+SAG groups. Scale bar = 200 µm. E) qRT‐PCR analysis for *Lox* and *Loxl2* genes in the tongue. F) Stiffness measurement procedure of mouse tongue specimens using nanoindentation. The measurement was performed on stromal regions identified using reference SR‐stained sections. G) The stiffness of mouse tongue tissues expressed in kilopascals (kPa). H) IHC for the presence of nuclear YAP‐expressing (YAP+) epithelial cells in the tongue and esophagus. The average percentage of nuclear YAP+ epithelial cells were quantified. Scale bars = 200 µm, 20 µm (inset). TF, transcription factor. I) qRT‐PCR analysis for the expression of YAP target genes (*Areg, Ctgf, Ptgs2*) in the tongue. J) IHC for nuclear GLI2‐expressing (GLI2+) epithelial cells in the tongue and esophagus. The average percentage of nuclear GLI2+ epithelial cells was quantified. Scale bars = 50 µm (tongue), 200 µm (esophagus), 20 µm (inset). The data are expressed as the mean ± standard error of mean (s.e.m.) from all individuals in each group. Statistical analysis involved one‐way analysis of variance (ANOVA) followed by post hoc Tukey's or Dunnett's test. Statistical significance was indicated by *p*‐values< 0.05, with varying numbers of asterisks denoting the levels of significance (*p <  0.05; **p <  0.01; ***p  <  0.001; and ****p <  0.0001). Representative images are shown for SR, IHC, and IF. K) Scatter plots showing the correlation between the relative collagen area and the percentage of nuclear GLI2+ epithelial cells in tongue and esophagus. Simple regression analysis was employed. L) Active interactions between epithelial cells and fibroblasts in the precancerous condition, involving the reciprocal exchange of chemical and mechanical signals. The SHH signaling originating from epithelial cells triggers the activation of fibroblasts, prompting their transformation into myofibroblasts responsible for generating collagens and enzymes, such as LOX, which contribute to the stiffening of extracellular matrix (ECM). Simultaneously, epithelial cells are responsive to the increased stiffness of the ECM, driving nuclear translocation of GLI2.

Both cancer cells and CAFs express Sonic Hh (SHH), one of the Hh ligands that acts as a messenger between cancer cells and CAFs in an autocrine and paracrine manner.^[^ ^19^ ^]^ Recent studies have reported that SHH is highly expressed in the dysplastic oral epithelium and OSCC,^[^ ^17^ ^]^ promoting cancer cell growth and invasion and governing interactions with stromal cells.^[^ ^19^, ^20^ ^]^ Particularly, CAFs expand in response to OSCC‐derived SHH and release Hh ligands themselves, reinforcing the influence of Hh signaling in tumor progression.^[^ ^19^ ^]^ However, most previous studies have shown correlations between pathological parameters of OSCC and SHH protein expression assessed by immunostaining in tissue specimens from patients with OSCC. To investigate whether Hh signaling involves in the pathogenesis of oral epithelial dysplasia, we established a mouse model of chronic tongue and esophageal tissue injury. Since the 4‐nitroquinoline‐1‐oxide (4‐NQO)‐induced model is known to reflect the multi‐stages of human carcinogenesis from dysplasia to invasive SCC in both tongue and esophagus,^[^ ^21^ ^]^ we treated 4‐NQO in combination with arecoline to mice via drinking water for 8 weeks (**Figure** **1**A). 4‐NQO is a precursor carcinogen mimicking tobacco, of which metabolites induce the formation of DNA adducts. Arecoline, an alkaloid extracted from areca (betel) nut, exhibits cytotoxic and genotoxic properties, promoting the development of OSCC when combined with 4‐NQO.^[^ ^22^ ^]^ After 8 weeks of exposure to 4‐NQO and arecoline (referred to as “NA”), mice were sacrificed, and tongue and esophageal tissues were immediately collected for analysis. By immunostaining and ELISA for SHH protein in mouse serum, we found that more epithelial cells expressed and released SHH in NA‐treated mice than in control mice that consumed plain water (referred to as “CTL”) (Figure 1B,C). In addition, the number of SHH‐expressing epithelial cells and SHH release were significantly upregulated in mice treated for 3 weeks via oral gavage with a smoothened agonist (SAG) that activates Hh signaling in a Ptch‐independent manner,^[^ ^23^ ^]^ in addition to NA, to hyperactivate Hh signaling after injury (referred to as “NA+SAG”) (Figure 1B,C).

Next, we assessed collagen accumulation in mouse tongue and esophagus, given that Hh signaling is implicated in tissue remodeling and fibrosis.^[^ ^24^ ^]^ Sirius Red staining showed that collagen deposition was upregulated after NA‐induced injury in both tongue and esophageal stroma, whereas only thin collagen fibers of the basement membrane were present in normal tissues (Figure 1D). Moreover, in the NA+SAG group, dense and thick collagen fibers were accumulated beneath the epithelium, accompanied by increased expression of collagen‐crosslinking enzymes, such as *Lox* and LOX‐like 2 (*Loxl2*) at the mRNA level (Figure 1D,E). Expecting that mechanical properties of the tissues, such as stiffness which is generally positively correlated with collagen deposition,^[^ ^25^ ^]^ might be altered after injury, we employed nanoindentation to measure tongue tissue stiffness. Results demonstrated an increase in tissue stiffness with elevated collagen accumulation and hyperactivation of Hh signaling (CTL: 5.1 ± 0.4, NA: 18.5 ± 0.8, NA+SAG: 40.8 ± 2.7 kPa) (Figure 1F,G).

As Hh signaling is known to activate myofibroblasts and CAFs that orchestrate the tumor microenvironment,^[^ ^26^ ^]^ we treated human gingival fibroblasts (HGF) with SAG, SHH, or TGF‐β as a positive control. Hyperactivation of Hh signaling induced by SAG or SHH treatment significantly increased the expression of α‐smooth muscle actin (α‐SMA), LOX, and LOXL2 proteins in HGF, even considering that untreated HGF was already in activated state as the protein levels were upregulated to similar extents after TGF‐β treatment (Figure S1A, Supporting Information). These changes were more pronounced when HGF was cultured on a soft substrate, where fibroblast activation is restricted^[^ ^27^ ^]^ (Figure S1B, Supporting Information). On the other hand, treatment with Vismodegib (GDC‐0449), an inhibitor of Hh signaling, suppressed the protein expression of α‐SMA, LOX, and LOXL2 in HGF (Figure S1A,B, Supporting Information). The effect of Hh signaling on fibroblast activation was validated in human dermal fibroblast (Figure S1C, Supporting Information). These results suggest that Hh signaling, particularly SHH derived from injured epithelial cells, may activate myofibroblasts during chronic injury, contributing to the establishment of a collagen‐rich, stiff matrix in the tongue and esophageal stroma (Figure 1L).

### 2.2 Increased Matrix Stiffness Promotes the Activation of Hedgehog Transcription Factor GLI via RhoA/ROCK/p‐MLC Axis

Cells that sense high matrix stiffness drive the nuclear localization of YAP, a mechanoresponsive transcriptional activator, through increased actin cytoskeleton tension.^[^ ^28^ ^]^ To explore whether epithelial cells adjacent to collagen‐rich stroma are mechanosensitive, we adopted a quantitative analysis of the nuclear expression of YAP as a marker of cellular mechanoresponsiveness. Results showed that epithelial cells surrounded by a higher density of collagen fibers displayed elevated YAP activity, as evidenced by nuclear localization of YAP and upregulation of its target genes, including *Areg*, *Ctgf*, and *Ptgs2* (Figure 1H,I).

GLIs, transcription factors downstream of Hh signaling, are not only activated by SHH signaling (canonical) but also regulated by other pro‐oncogenic pathways, such as EGF, NF‐κB, RAS/MEK/AKT, TGF‐β, and TNF‐α/mTOR. This non‐canonical, Hh ligand‐ and Smo‐independent activation of GLI explains the higher aggressiveness of cancers harboring non‐canonical Hh signaling and the acquired resistance to Smo inhibitors in cancer therapy.^[^ ^29^ ^]^ These findings prompted us to investigate whether GLI2 activity is also regulated by matrix stiffness. Similar to YAP, the nuclear GLI2‐expressing epithelial cells became substantially increased in both the tongue and esophagus of NA‐treated mice compared to CTL mice, and the population of these cells was significantly higher in NA+SAG‐treated mice than in NA‐treated mice (Figure 1J). Also, the numbers of nuclear GLI2‐positive epithelial cells positively correlated with collagen deposition in both tongue and esophageal tissues (Figure 1K), suggesting that GLI2 might be mechanically activated by sensing ECM stiffness in a subpopulation of epithelial cells (Figure 1L).

**Figure 2:**
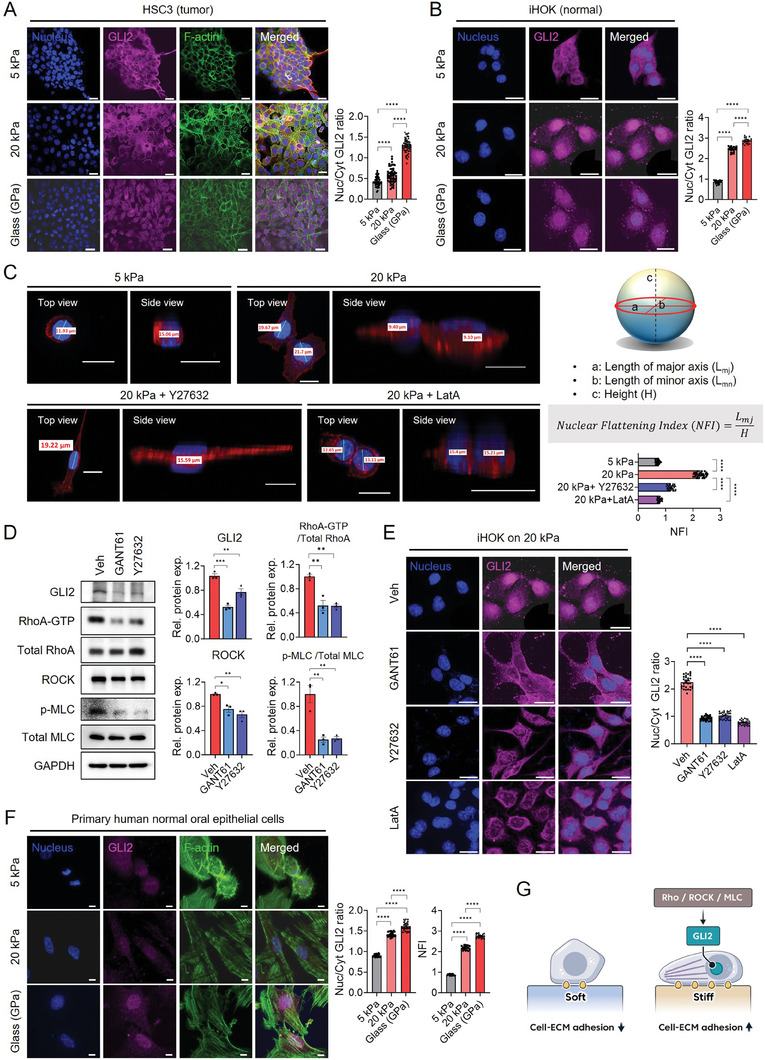
Increased matrix stiffness promotes GLI2 activation through RhoA/ROCK/p‐MLC axis‐mediated actin cytoskeletal tension. A) Representative confocal images depicting nuclei (blue), GLI2 (magenta), and F‐actin (green) in HSC3 cells cultured on soft (5 kPa), stiff (20 kPa) hydrogels, or glass (≈ GPa) substrate. The nuclear to cytoplasmic ratios (Nuc/Cyt) of GLI2 expression were quantified. Scale bar = 20 µm. B) Representative confocal images and quantification of GLI2 expression in iHOK cells cultured on 5 or 20 kPa hydrogels, or glass substrate. Scale bar = 20 µm. C) The nuclear flattening index (NFI) was calculated by dividing the length of the major axis (a) of a spheroid‐shaped nucleus by its height (c), where a higher NFI value signifies greater nuclear flattening. Representative images of HSC3 cells stained for F‐actin (red) and nuclei (blue) were shown under defined conditions. Scale bar = 20 µm. D) Immunoblots showing GLI2, RhoA‐GTP, total RhoA, ROCK, phosphorylated MLC (p‐MLC), total MLC, and GAPDH as a loading control in HSC3 cells treated with GANT61 or Y27632 compared to the vehicle (Veh). E) IF images of GLI2 (magenta) and DAPI (blue) staining in the presence of the inhibitors GANT61, Y27632 or LatA relative to the Veh. Scale bar = 20 µm. The mean ± s.e.m. results are presented. Statistical analysis involved one‐way ANOVA followed by post hoc Tukey's or Dunnett's test for multiple group comparisons (*p  <  0.05; **p  < 0.01; ***p <  0.001; and ****p  <  0.0001). F) Representative confocal images depicting GLI2 (magenta), F‐actin (green), and DAPI (blue) staining in primary oral epithelial cells isolated from human normal gingival mucosal tissues cultured on either 5 or 20 kPa hydrogels, or glass substrates. Analysis of GLI2 expression and nuclear flattening was conducted as illustrated above. Scale bar = 10 µm. G) Enhanced matrix stiffness promotes the nuclear translocation of GLI2 within epithelial cells through the orchestration of actin polymerization and activation of the Rho/ROCK/MLC signaling pathway. This activation results in heightened tension within the actomyosin cytoskeleton. The reinforcement of cell‐ECM adhesions plays an important role in sensing the elevated matrix stiffness and mediating the mechanotransduction.

To examine the effect of matrix stiffness on GLI2 nuclear translocation, we developed RGD‐functionalized MeHA hydrogels with either low (5 kPa; similar to CTL tongue) or high (20 kPa; similar to NA‐treated tongue) stiffness and utilized them and glass bottom dishes (≈GPa) as cell culture platforms (Figure S2A,B, Supporting Information). In this study, we mainly utilized HSC3 (a human OSCC cell line) and immortalized human oral keratinocytes (iHOKs; a representative normal cell line). While iHOK cells exhibit slow growth and limited migratory capacity, they were chosen specifically to investigate early events in non‐tumor epithelial cells during cancer development, particularly focusing on the mechanoresponsiveness of epithelial cells. As a counterpart, HSC3 cells were selected due to their widespread use in numerous studies and their high tumorigenic potential, making them a suitable model for investigating cancer‐related processes, such as EMT. Initially, we validated that an increase in substrate stiffness led to elevated nuclear‐to‐cytoplasmic YAP ratio in both HSC3 and iHOKs (Figure S2C,D, Supporting Information). In addition, cell morphology, characterized by spreading area, circularity and aspect ratio, was affected by the stiffness, indicating the sensitivity and responsiveness of both cell lines to substrate stiffness. Of note, the nuclear‐to‐cytoplasmic GLI2 ratio was also upregulated, similar to YAP, as the stiffness increased, in both HSC3 and iHOK (**Figure** **2**A,B). We investigated the frequency of PC, where the Hh receptor Ptch is located, in cells arrested at the G0/G1 phase of the cell cycle by serum starvation. A recent study has shown that PC is lost in the potentially pre‐malignant oral epithelium (i.e., leukoplakia) and OSCC compared to normal oral mucosal epithelium.^[^ ^30^ ^]^ In line with the previous report, when cultured on glass bottom dishes, we observed minimal PC in five different OSCC cell lines to validate the common occurrence of PC loss in cancer cells, including HSC3 (0.9 ± 0.2%), HSC2 (1.9 ± 0.2%), MC3 (0%), HN22 (2.3 ± 0.4%), Ca 9.22 (0%), whereas approximately a quarter of iHOKs exhibited PC (25 ± 0.2%) (Figure S3A, Supporting Information). Interestingly, we noticed a significantly higher presence of PC in HSC3 cells when cultured on relatively softer surfaces (Figure S3B, Supporting Information). Previous studies have shown that GLI2 becomes constitutively active in concomitance with the loss of PC, even accelerating tumor formation.^[^ ^31^ ^]^ To examine whether PC disassembly influences GLI2 activation, we treated iHOKs with a non‐toxic dose of tubacin (Figure S4A, Supporting Information), a selective inhibitor of α‐tubulin deacetylating activity of Histone deacetylase 6 (HDAC6) without affecting histone acetylation, gene expression, or cell cycle,^[^ ^32^ ^]^ to protect cells from HDAC6‐mediated PC resorption under high stiffness conditions. Despite culturing cells on glass bottom dishes, tubacin treatment resulted in approximately twofold increase in ciliated iHOKs (Figure S4B, Supporting Information). Surprisingly, nuclear translocation of GLI2 was markedly suppressed after tubacin treatment, regardless of substrate stiffness (Figure S4C, Supporting Information). We confirmed also a significant decrease in filamentous actin (F‐actin) intensity in iHOKs after tubacin treatment (Figure S4D, Supporting Information), consistent with the previous report that tubacin inhibited HDAC6‐induced actin polymerization in retinal pigment epithelial cells.^[^ ^32^ ^]^

To further validate the mechanical activation of GLI2 under high substrate stiffness, we transduced HSC3 cells with lentiviral shRNA targeting the *Smo* gene (shSMO) to knockdown Smo expression, along with a negative control shRNA (shCTL), and then cultured them on either soft or stiff substrates. Interestingly, we observed that GLI2 maintained its nuclear localization under high stiffness conditions even in the absence of Smo expression (Figure S5, Supporting Information). The nuclear‐to‐cytoplasmic ratio of GLI2 expression remained significantly higher in shSMO‐transduced cells on stiff substrates compared to soft substrates, although slightly lower than in shCTL‐transduced cells on stiff substrates. This difference between shSMO‐ and shCTL‐transduced cells on stiff substrates may reflect the proportion of Smo‐dependent canonical Hh signaling contributing to GLI2 activation. Without additional Hh‐activating factors in the culture medium, the highly maintained nuclear localization of GLI2 appeared to be solely dependent on matrix stiffness and independent on Smo, suggesting a non‐canonical activation of GLI2.

Since actomyosin‐generated cytoskeletal tension induces nuclear flattening, which is strongly correlated with YAP nuclear import,^[^ ^25^ ^]^ we analyzed nuclear flattening index (NFI) based on 3D DAPI staining images of HSC3 cells cultured on soft or stiff substrates (Figure 2C). The NFI values of HSC3 cells on stiff substrates were significantly higher than those on soft substrates (Figure 2C), indicating an increased actin cytoskeletal tension on stiff substrates. YAP nuclear localization is also regulated by its phosphorylation state, which is suppressed by actomyosin contractility. To determine whether GLI2 nuclear localization is also similarly regulated by actomyosin contractility which is governed by RhoA/ROCK/MLC axis,^[^ ^33^ ^]^ we treated HSC3 cells in tissue culture plates with Y27632 (a ROCK inhibitor), Latrunculin A (LatA, an actin polymerization inhibitor), or GANT61 (a GLI2 inhibitor) as a positive control at non‐toxic doses (Figure S6, Supporting Information). Treatment of Y27632 decreased expression of active GTP‐bound RhoA, ROCK, and phosphorylated MLC (p‐MLC) proteins, evidencing actomyosin contractility was successfully inhibited in treated cells (Figure 2D). In Y27632‐treated cells, the NFI values were significantly reduced and the expression and nuclear translocation of GLI2 were diminished, even on stiff substrates (Figure 2C–E). These results were reproduced with LatA treatment (Figure 2C and E). Unexpectedly, we observed that protein levels of active RhoA, ROCK, p‐MLC were significantly downregulated after GANT61 treatment (Figure 2D), implicating a potential feedback loop between GLI2 and RhoA/ROCK/MLC signaling. Notably, our findings observed in HSC3 and iHOK cells were successfully replicated in primary oral epithelial cells isolated from human normal gingival mucosal tissues. Specifically, we observed a significant increase in nuclear translocation of GLI2 as stiffness increased, accompanied by a significant increase in nuclear flattening (Figure 2F and Figure S7, Supporting Information). Together, these results suggest that GLI2 might act a mechanotransductive transcription factor, activated in response to high stiffness through actin polymerization and the RhoA/ROCK/MLC axis‐mediated actin cytoskeletal tension (Figure 2G).

### 2.3 Mechanically Activated Hedgehog Signaling Promotes Proliferation, Epithelial‐to‐Mesenchymal Transition, Migration, and Invasion

**Figure 3:**
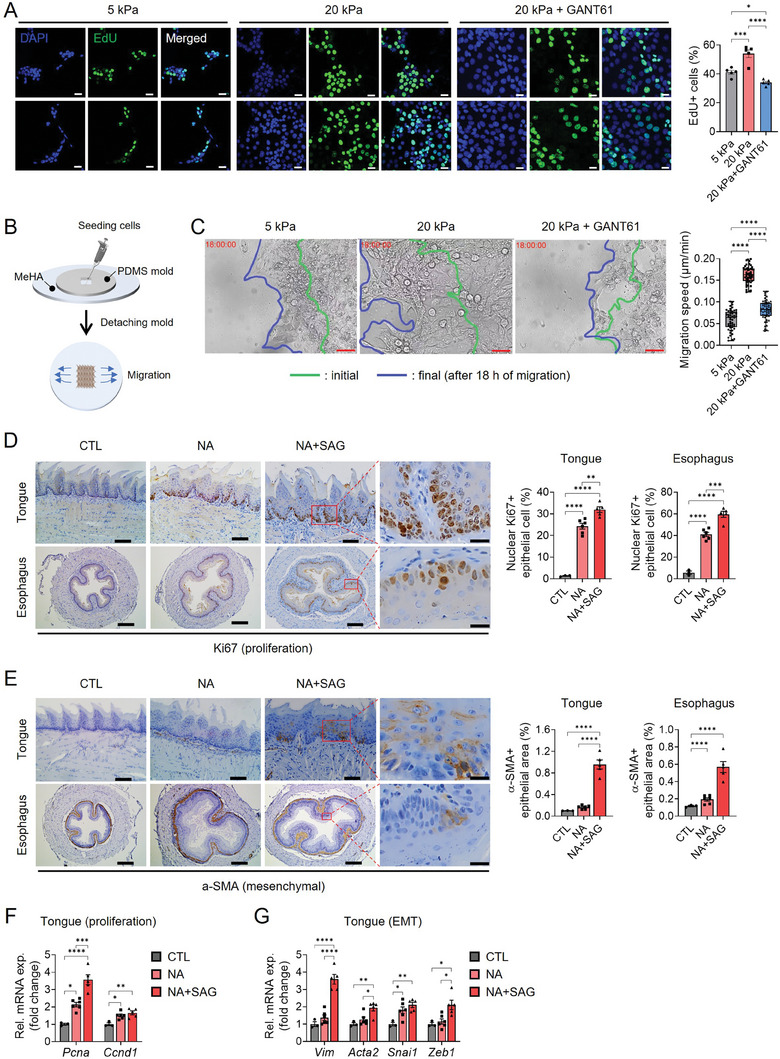
Epithelial cells acquire GLI2‐dependent proliferative and migratory capabilities during tongue/esophageal epithelial dysplasia. A) Confocal images and quantification of EdU (green) stained nuclei in HSC3 cells cultured on 5 or 20 kPa hydrogels, with or without GANT61 (15 × 10^−6^ m) treatment for 24 h. Scale bar = 20 µm. B) A schematic illustration demonstrating collective migration assay involving confined cell seeding on MeHA hydrogels with different stiffness levels. PDMS, polydimethylsiloxane. C) Brightfield images illustrating the migration of HSC3 cells on MeHA hydrogels with different stiffness levels, with or without GANT61 treatment. The images display the initial (green lines) and final (blue lines) positions of cells after 18 h of migration. The migration speed was measured for each condition and is represented as box and whisker plots, displaying all data points from the minimum to the maximum values. Scale bar = 100 µm. D) IHC for nuclear Ki67+ epithelial cells and the percentage in the tongue and esophagus of CTL, NA, and NA+SAG mice. Scale bar = 200 µm, 20 µm (inset). E) IHC for α‐SMA+ epithelial cells and the area in the tongue and esophagus of the mice. Scale bar = 200 µm, 20 µm (inset). qRT‐PCR analysis for the expression of F) proliferation markers (*Pcna, Ccnd1*) and G) mesenchymal markers (*Vim, Acta2, Snail1, Zeb1*). The mean ± s.e.m. results are displayed. Statistical analysis involved one‐way ANOVA followed by post hoc Tukey's or Dunnett's test for multiple group comparisons (*p  < 0.05; **p <  0.01; ***p <  0.001; and ****p  <  0.0001). Representative images are shown.

To determine the functional role of mechanically activated Hedgehog signaling in epithelial cells, we compared HSC3 cells cultured on soft or stiff MeHA hydrogels before and after treatment with the pharmacological GLI2 inhibitor, GANT61. After confirming that upregulated GLI2 mRNA expression in HSC3 cells on a stiffer substrate was downregulated by GANT61 treatment (Figure S8A, Supporting Information), we found that GLI2 inhibition significantly downregulated mRNA expression of cell proliferation markers (*Cyclin D1* (*CCND1*), *CCNB1, Cyclin‐Dependent Kinase 2* (*CDK2*)) which was upregulated on stiffer substrate (Figure S8B, Supporting Information). Subsequently, we performed EdU staining, which revealed a significant increase in EdU incorporation in HSC3 cells when cultured on stiffer substrate (**Figure** **3**A). Additionally, the inhibition of GLI2 with GANT61 resulted in a reduction in the number of EdU‐positive cells (Figure 3A), suggesting that GLI2 activation fosters cell proliferation.

EMT is a process in which epithelial cells acquire the characteristics of migratory and invasive mesenchymal cells.^[^ ^34^ ^]^ We found the expression of mesenchymal markers (*ACTA2* that encodes α‐SMA protein, *Matrix Metalloproteinase 2* (*MMP2*), *MMP9*, *SOX10*), *TGF‐β1* (a strong promoter of EMT), and its receptor *TGF‐βR1* was significantly downregulated by GLI2 inhibition (Figure S8B, Supporting Information). A collective migration assay on the MeHA hydrogels showed that HSC3 cells migrate significantly faster on stiffer substrate (Figure 3B,C and Videos S1 and S2, Supporting Information), indicating that the migration speed of HSC3 cells is affected by substrate stiffness. The increased migration speed of cells on stiff substrates was abrogated by GLI2 inhibition to the extent comparable to that on soft substrate (Figure 3C and Video S3, Supporting Information). The migratory activity may be governed by a GLI2‐mediated transcriptomic program, as evidenced by the significant downregulation of genes targeted by GLI2 (*E2F1*, *BCL2*, *IL1‐β*, *IL6*, *IL7*, *FNDC1*, *ARHGEF16*)^[^ ^35^ ^]^ upon GLI2 inhibition (Figure S7B, Supporting Information). Notably, the Rho guanine nucleotide exchange factor 16 (ARHGEF16) is known to activate Cdc42 and Rac1 proteins, two Rho GTPases that play pivotal roles in cell migration, working together with RhoA‐dependent contractility.^[^ ^36^ ^]^ This suggests a potential involvement of Cdc42 and Rac1 in GLI2‐dependent cell migration, further supported by the observation that protruding activity on leader cells appeared to be inhibited after GANT61 treatment (Video S3, Supporting Information). Furthermore, transwell migration and invasion assays consistently demonstrated a substantial reduction in the mobility and invasiveness of HSC3 cells following GANT61 treatment (Figure S8C,D, Supporting Information). These findings collectively establish that mechanically activated GLI2 is responsible for mechanosensitive regulation of EMT through which oral epithelial cells eventually acquire migratory and invasive capabilities to penetrate the basement membrane and ECM during the initiation of invasive SCC.

### 2.4 Hedgehog Signaling Promotes the Progression of Oral/Esophageal Epithelial Dysplasia in a Mouse Model

In our mouse model of NA‐induced chronic tissue injury, hematoxylin and eosin (H&E) staining revealed a significant increase in tongue and esophageal epithelial thickness in mice subjected to NA treatment compared to CTL (Figure S9, Supporting Information). Immunostaining further demonstrated that basal epithelial cells, which highly expressed SHH, GLI2 and YAP (Figure 1B,H,J), were also positive for Ki67, a marker of cell proliferation, in NA‐treated mice (Figure 3D). Remarkably, the Ki67‐positive proliferating epithelial cells were significantly more abundant in the NA+SAG group (Figure 3D). Moreover, subpopulations of tongue and esophageal epithelial cells in the NA and NA+SAG group displayed α‐SMA and Vimentin expression, while they lost the expression of E‐cadherin, an epithelial marker (Figure 3E and Figure S10A,B, Supporting Information). On the other hand, α‐SMA and Vimentin‐expressing epithelial cells were scarcely observed in tongue and esophagus of a CTL group (Figure 3E and Figure S10A, Supporting Information). This implies that EMT might occur through mechanical hyperactivation of GLI2 signaling following injury. Accordingly, expressions of proliferation markers, such as *Pcna* and *Ccnd1*, and mesenchymal markers, such as *Vimentin* (*Vim*), *Acta2*, *Snail* (*Snai1*), *Zeb1*, *Fibronectin* (*Fn1*), *Mmp2*, *Mmp9*, *Sox10*, *Itgb1*, *Itgb2*, and *Tgf‐β induced* (*Tgfbi*) were significantly upregulated at the mRNA level in tongue tissues of NA+SAG‐treated mice compared to the CTL mice (Figure 3F,G and Figure S10C, Supporting Information). Although the expression of proliferation markers was also upregulated in NA‐treated mice compared to CTL mice, the majority of proliferation and mesenchymal marker genes examined exhibited even higher expression levels in the NA+SAG‐treated mice than in the NA‐treated mice (Figure 3F, G). This indicates that the combined effects of increased Hh signaling activity and enhanced ECM stiffness synergistically surpass the signaling threshold necessary for EMT. The findings highlight that both factors act in concert, creating a more potent and conducive environment for the development of dysplastic growth in epithelial cells (i.e., precancerous condition) following chronic injury.

### 2.5 Spatial Transcriptomics Reveals Transcriptional Architecture at the Epithelial–Stromal Interface during the Precancerous Condition

**Figure 4:**
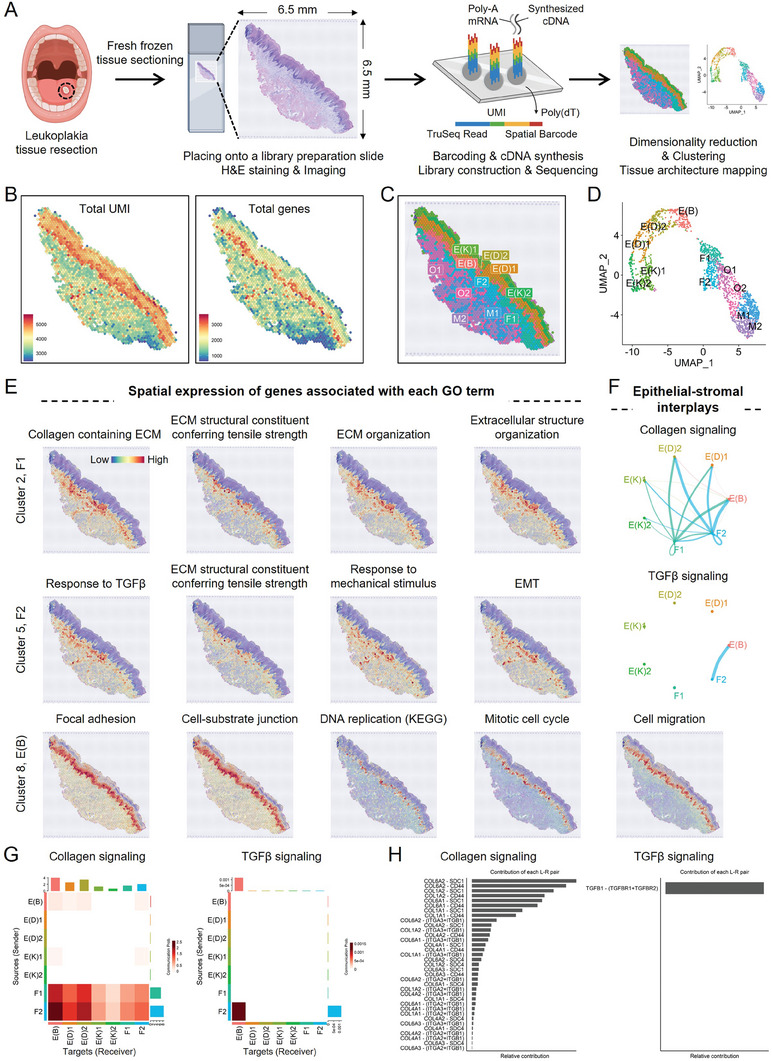
Spatial transcriptomics analysis reveals the mechanobiological dialogues between epithelial cells and fibroblasts during a precancerous condition in the human tongue. A) The diagram illustrates the workflow of the spatial transcriptomics analysis conducted on a cryosection of a leukoplakia lesion in the human tongue. B) The Visium array spots on the tissue section are color‐coded based on the number of normalized unique molecular identifiers (UMIs, left) or total genes (right) in the dataset. C) The Visium array spots are colored based on the clustering assignments generated from the dataset. A total of 11 clusters were identified, including E(K)1 and E(K2) (epithelial keratinocyte clusters 1 and 2, respectively), E(D)1 and E(D)2 (differentiating epithelial cell clusters 1 and 2), E(B) (basal epithelial cell cluster), F1 and F2 (fibroblast clusters 1 and 2), M1 and M2 (muscle cell clusters 1 and 2), and O1 and O2 (unspecified, others 1 and 2). D) The spatial transcriptomics spots are visualized using a UMAP embedding, with colors representing the cluster assignments. E) The average standardized expression of annotated genes associated with gene ontology (GO) terms exhibiting spatially coherent expression patterns within each cluster is displayed. The expression levels are depicted using a color gradient, ranging from low (blue) to high (red). F–H) Cell‐cell communication analysis reveals significant collagen and TGF‐β signaling between epithelial cells and fibroblasts in the dataset. F) The circle plot illustrates the communication score between interacting cell clusters, with line thickness indicating the strength of communication. G) The heatmap depicts the sender‐receiver interaction matrix, where rows and columns represent sources and targets, respectively. The bar plots on the right and top represent the total outgoing and incoming interaction scores, respectively. H) The relative contribution of ligand (L)–receptor (R) pairs involved in the communication is shown. SDC, syndecan; ITGA, integrin subunit alpha; ITGB, integrin subunit beta.

Abundant α‐SMA‐positive CAFs are located near SHH‐expressing cancer cells, particularly in invasive cancer,^[^ ^19a^ ^]^ suggesting that CAFs assist cancer cell invasion upon recruitment by SHH signaling. Thus, we further sought to explore the significance of physicochemical interactions between epithelial cells and the neighboring stroma, comprising mainly fibroblasts and ECM, in the development of a microenvironment favorable for tumorigenesis. For this purpose, we conducted capture probe‐based spatially resolved transcriptomics using the 10 × Genomics Visium platform on frozen tongue tissue sections from a patient with leukoplakia, a potentially malignant disorder (**Figure** **4**A). The tissue section harbored a pathologist‐confirmed dysplasia lesion at its center. The Visium dataset provided transcriptomes for 1854 barcoded array spots, encompassing 18103 unique genes. We observed a range of 2438 to 5685 transcripts (unique molecular identifiers, UMIs) and 645 to 3652 unique genes per spot (Figure 4B, Supporting Information). Notably, the array spots within the epithelial region contained a higher number of UMIs and genes compared to the rest of the tissue. Specifically, the basal epithelial cells, which establish direct physical contact with the stroma, exhibited the highest number of detected genes (Figure 4B, right). We identified 11 distinct clusters and confirmed their assignments by projecting them back onto tissue coordinates (Figure 4C) and uniform manifold approximation and projection (UMAP) embeddings for each spot (Figure 4D). This validation process ensured that the spatial patterns in the data faithfully reflected the tissue histology. Additionally, we verified the cluster assignments by examining the expression of marker genes specific to epithelial cells (*CDH1*, *ADH7*, *KRT15*), fibroblasts (*ACTA2*, *COL1A1*, *COL1A2*, *COL6A2*), and muscle cells (*DES*, *ENO3*, *FLNC*, *TTN*) in UMAP (Figure S11A–D, Supporting Information) and tissue coordinates (Figure S12, Supporting Information). The localized expression of these marker genes aligned precisely with the expected regions, confirming the accuracy of the cluster assignments.

Next, we conducted human gene ontology (GO) enrichment analysis and Kyoto Encyclopedia of Genes and Genomes (KEGG) pathway analysis using automatically identified marker genes from each cluster (*p* < 0.05, log_2_FC > 0; Table S1, Supporting Information). Our analysis revealed significant enrichment of GO terms and pathways associated with ECM structural organization and mechanical stimulus in F1 (fibroblasts, cluster 2) and F2 (fibroblasts, cluster 5) (*p* < 0.05; Table S2, Supporting Information). Notably, molecular functions and biological processes linked to cells cultured on a stiff substrate, such as Focal adhesion, Cell‐substrate junction, DNA replication, Mitotic cell cycle, and Cell migration, were enriched in E(B), basal epithelial cells (cluster 8). When we applied the mean expression of marker genes associated with these GO terms to the tissue coordinates, we observed spatial expression patterns related to tissue structure (Figure 4E), reminiscent of our findings from the mouse model of tongue/esophageal epithelial dysplasia (Figures 1 and 3). Analysis of the ligand‐receptor network revealed strong Collagen and TGF‐β signaling between fibroblasts and epithelial cells in leukoplakia, particularly between F2 and E(B) (Figure 4F–H). Notably, collagen genes (*COL1A1*, *COL1A2*, *COL6A1*, *COL6A2*) and ECM receptor genes (*CD44*, *SDC1*, *ITGA2*, *ITGA3*) were predominantly expressed in F2 and E(B) cluster, respectively (Figures S11C,S12B, and S13, Supporting Information). Similarly, *TGF‐β1* and its receptor, *TGF‐βR1*, exhibited expression patterns consistent with collagen genes and integrins. In addition, the expression of ROCK1 was higher in F2 and E(B), suggesting that F2 may represent contractile, ECM‐remodeling fibroblasts, while E(B), which also exhibited high expression of NOTCH1 (a mechanosensitive receptor that promotes cell proliferation^[^ ^37^ ^]^) and KRT15 (a basal cell marker that is upregulated in SCC^[^ ^38^ ^]^), may undergo dysplastic growth within a stiff microenvironment (Figures S11B,S12A, and S13, Supporting Information). It is noteworthy that gene sets highly expressed in the F1 and F2 clusters of leukoplakia, associated with ECM structural organization and mechanical stimulus, were scarcely expressed near the epithelium in normal human oral mucosal tissue (Figure S14, Supporting Information). Additionally, gene sets exclusively highly expressed in the E(B) cluster of leukoplakia and associated with focal adhesion, cell–substrate junction, DNA replication, and mitotic cell cycle were not enriched in epithelial cell clusters of normal tissue. These findings together suggest that there were fewer mechanical interactions between the epithelial cells and fibroblasts (or ECM) in normal tissue compared to leukoplakia.

### 2.6 Single‐Nucleus RNA‐Seq Demonstrates That GLI2‐Expressing Epithelial Cells Acquire the Properties of Cancer

**Figure 5:**
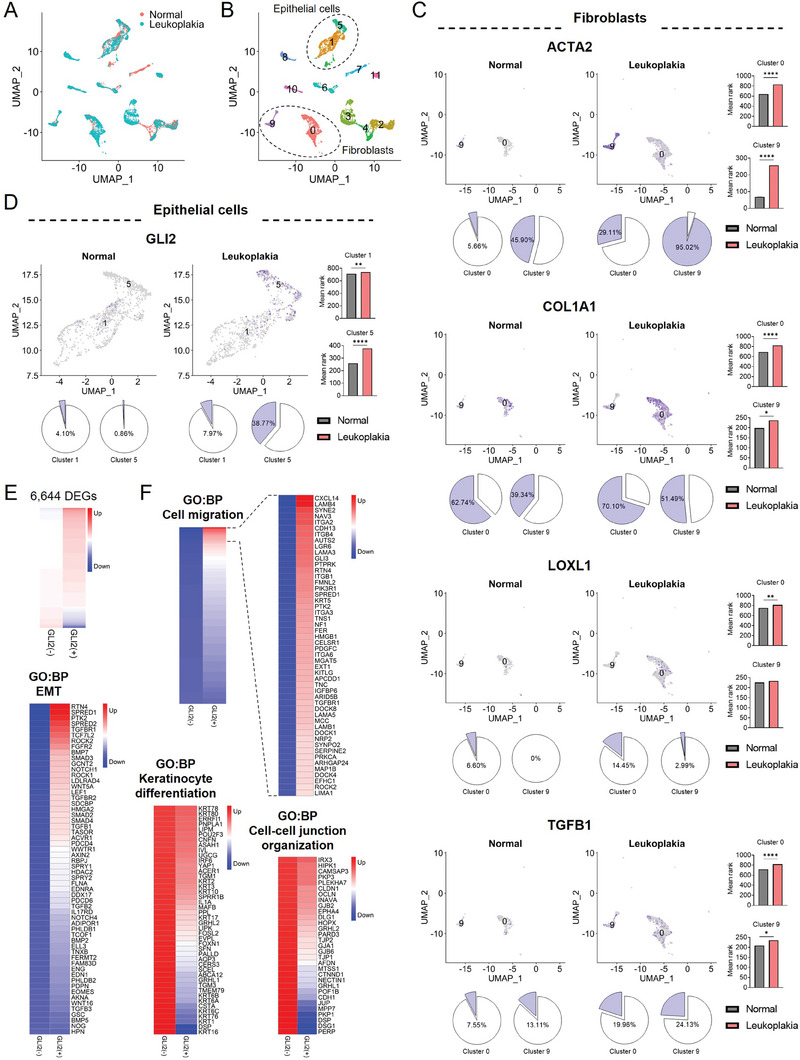
Single‐nucleus RNA sequencing (snRNA‐seq) shows that epithelial cells expressing GLI2 acquire specific gene expression patterns and alterations in molecular signatures associated with cancer. A) The integrated UMAP plot combines the snRNA‐seq datasets of normal and leukoplakia samples, displaying the cellular landscape in a reduced‐dimensional space. B) The snRNA‐seq cluster assignments are visualized in UMAP space, indicating the different cell clusters present in the dataset. C) The expression of myofibroblast‐related genes (*ACTA2*, *COL1A1*, *LOXL1*, *TGFB1*) is shown for fibroblast clusters (cluster 0 and 9) in the snRNA‐seq dataset. The pie charts represent the percentage of positive nuclei for each gene within the respective cluster. The bar plots indicate the mean rank± s.e.m. for gene expression. Statistical significance was determined using the Mann‐Whitney U test, with a *p*‐value threshold of less than 0.05. D) The expression of GLI2 is visualized in the epithelial cell clusters (cluster 1 and 5) in the snRNA‐seq dataset. E) The heatmap visualizes the different gene profiles between GLI2‐positive and GLI2‐negative epithelial cells in leukoplakia (*p* < 0.05). DEG, differentially expressed gene. F) Heatmaps depict the expression of genes associated with GO terms enriched in GLI2‐expressing epithelial cells. The color gradient represents the expression levels, ranging from downregulated (blue) to upregulated (red) expression specifically in GLI2‐expressing cells.

To compare the transcriptional profiles of human leukoplakia and normal oral mucosal tissue, we conducted single‐nucleus RNA sequencing (snRNA‐seq) on nuclei extracted from both sample types. After quality control and filtering steps, we obtained a dataset consisting of transcriptomes for 9748 individual nuclei (**Figure** **5**A), with an average of 3368 UMIs and 1779 unique genes per nucleus. Using dimensionality reduction and clustering techniques, we identified 12 distinct clusters (Figure 5A,B). For further analysis, we focused on epithelial cells (clusters 1, 5) and fibroblasts (clusters 0, 9) that expressed genes specific to their respective cell types in the dataset (Figure S15, Supporting Information). Since the number of individual nuclei of normal and leukoplakia tissues differed, we compared the proportion of positive nuclei for genes of interest in total nuclei and the mean rank of gene expression per cluster. The number of nuclei per cluster, as summarized in Table S3, Supporting Information, showed a significant accumulation of *ACTA2*‐, *COL1A1*‐, *LOXL1‐*, and *TGFB1*‐expressing fibroblasts in leukoplakia tissue, in contrast to normal tissue (Figure 5C and Table S3, Supporting Information). These findings consistently support the activation of fibroblasts into myofibroblast‐like cells involved in ECM production and remodeling during the precancerous condition. Furthermore, comparing epithelial cell clusters revealed that GLI2 expression was significantly upregulated in a higher proportion of epithelial cells in leukoplakia tissue compared to normal tissue (Figure 5D). To further characterize the GLI2‐expressing epithelial cells in leukoplakia, we re‐analyzed the gene profiles of epithelial cells based on *GLI2* expression and identified 5491 upregulated and 1153 downregulated genes in GLI2‐expressing cells compared to cells without GLI2 expression (*p* <  0.05, Figure 5E and Table S4, Supporting Information).

Subsequent analyses of GO enrichment and KEGG pathway revealed that GLI2‐expressing cells exhibited a loss of gene expression associated with keratinocyte differentiation, cell‐cell junction organization, cell‐cell adhesion, cadherin binding, and tight junction, while gaining gene expression associated with cell migration, EMT, TGF‐βR signaling pathway, integrin‐mediated signaling pathway, ECM organization, cell‐substrate interaction, focal adhesion, actin cytoskeleton organization, Hedgehog signaling pathway, pathways in cancer, and cell cycle (Figure 5F **;** Figure S16 and Table S5, Supporting Information). These findings imply that GLI2‐expressing cells acquire properties resembling cancer cells. When visualizing the expression of several EMT‐related genes known to be upregulated in cancer and GLI2‐expressing cells from our snRNA‐seq dataset in a UMAP, we observed significant upregulation of genes such as *AXIN2*, *FGFR2*, *HMGA2*, *NOTCH1*, *ROCK1*, *SMAD2*, *TGFB1*, and *TGFBR1* in the epithelial cells of leukoplakia tissue compared to normal tissue (Figure S17, Supporting Information). This validation provides further evidence that the gene expression patterns of GLI2‐expressing cells are consistent with those observed in cancer cells, further supporting their potential role in the development of precancerous lesions. Additionally, in line with the findings from spatial transcriptomics data analysis, the snRNA‐seq data confirmed the presence of collagen signaling between fibroblasts and epithelial cells (Figure S18, Supporting Information). This indicates that fibroblast‐derived collagen‐rich ECM triggers pro‐tumorigenic signaling in GLI2‐expressing epithelial cells.

### 2.7 Extracellular Matrix Softening by Inhibiting Collagen Crosslinking Slows the Tumor Progression in Mice

**Figure 6:**
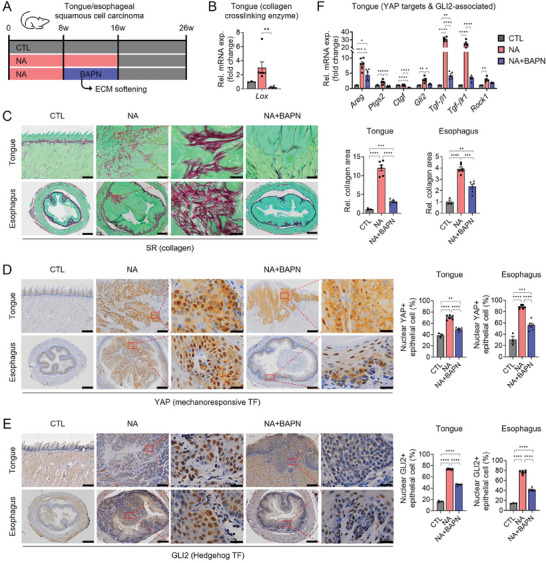
Extracellular matrix (ECM) softening by inhibiting collagen crosslinking reduces collagen contents and inhibits the activation of YAP and GLI2. A) The in vivo study design and timeline schematically illustrating a 16‐week exposure to NA with (*n* = 6) or without (*n* = 6) additional administration of BAPN (100 mg kg^−1^ day^−1^, twice a week), a LOX inhibitor, via intraperitoneal injection during the last 8 weeks, followed by a 10‐week developmental period without any further exposure to NA. The control (CTL) group consisted of age and sex‐matched mice (*n* = 3) that consumed plain water throughout the experimental period. B) qRT‐PCR for the *Lox* gene in the tongue of CTL, NA, and NA+BAPN mice. C) SR staining and quantification of the relative collagen area in the tongue and esophageal tissue sections of the mice. Scale bar = 200 µm, 20 µm (inset). D,E) IHC for nuclear YAP+ (D) and nuclear GLI2+ (E) epithelial cells in the tongue and esophagus of CTL, NA, and NA+BAPN mice. Scale bar = 200 µm, 20 µm (inset). TF, transcription factor. F) qRT‐PCR for the YAP target genes (*Areg*, *Ptgs2*, *Ctgf*), *Gli2*, and GLI2‐associated genes (*Tgf‐β1*, *Tgfbr1*, *Rock1*). The data presented represent the mean ± s.e.m. from all individuals within each group. Statistical analysis involved one‐way ANOVA followed by post hoc Tukey's or Dunnett's test (*p  <  0.05; **p <  0.01; ***p < 0.001; and ****p  <  0.0001). Representative images are shown for SR and IHC.

To investigate the potential of mitigating ECM stiffness to hinder the progression of tongue/esophageal SCC, we generated a mouse model of tongue/esophageal SCC by administering the carcinogens 4‐NQO and arecoline to mice for 16 weeks, followed by an additional 10‐week period without exposure to the carcinogens (referred to as “NA” group) (**Figure** **6**A). In half of the mice receiving the carcinogens, we intraperitoneally administered the irreversible LOX inhibitor β‐aminopropionitrile (BAPN) during the last 8 weeks of the carcinogen exposure period (referred to as “NA+BAPN” group) (Figure 6A). LOX is an enzyme involved in collagen crosslinking, contributing to ECM organization and stiffness. Treatment with BAPN disrupts LOX activity, leading to reduced ECM content and tumor stiffness.^[^ ^6^ ^]^ We observed a significant downregulation of *Lox* mRNA in tongue tissues of mice in the NA+BAPN group compared to the NA group (Figure 6B). Furthermore, Sirius Red staining revealed considerable collagen accumulation around tongue and esophageal tumors of the NA group compared to age‐ and sex‐matched control mice (referred to as “CTL”) (Figure 6C). However, in the NA+BAPN group, collagen accumulation was significantly reduced in tongue and esophageal tumors, confirming the successful reduction of collagen content and ECM stiffness with BAPN treatment. Immunohistochemistry for nuclear YAP expression demonstrated an increased number of epithelial cells with strong nuclear YAP expression in the NA group compared to the CTL group (Figure 6D). However, the number of cells expressing nuclear YAP significantly decreased in the NA+BAPN group as LOX activity and subsequent collagen accumulation decreased (Figure 6D). Similarly, there was a significant reduction in the population of epithelial cells expressing nuclear GLI2 in the tongue and esophagus of the NA+BAPN group compared to the NA group. In the NA group, approximately 74–77% of epithelial cells in the tongue (73.6 ± 0.9%) and esophagus (76.6 ± 3.4%) expressed GLI2 in the nucleus, whereas the population of epithelial cells expressing nuclear GLI2 was significantly reduced to 46.6 ± 1.6% in the tongue and 41.6 ± 3.3% in the esophagus of the NA+BAPN‐treated mice (Figure 6E). These changes were accompanied by the altered expression of YAP target genes (*Areg*, *Ptgs2*, *Ctgf*), *Gli2*, and GLI2‐associated genes (*Tgfb1*, *Tgfbr1*, *Rock1*) (Figure 6F). These results indicate that ECM softening inhibited the mechanical activation of GLI2.

**Figure 7:**
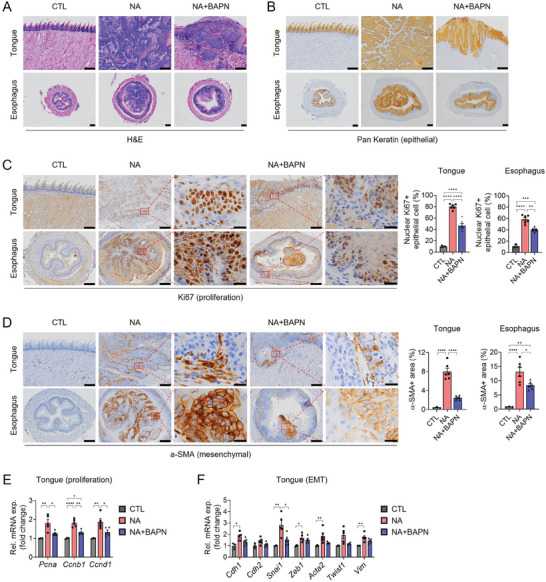
ECM softening slows the progression of tongue/esophageal SCC in mice. A) Hematoxylin and eosin (H&E) staining in the tongue and esophageal tissue sections of CTL, NA, and NA+BAPN mice. Scale bar = 200 µm. B) IHC for pan keratin in the tongue and esophageal tissue sections of the mice. Scale bar = 200 µm. IHC for C) Ki67 and D) α‐SMA and quantification of nuclear Ki67+ epithelial cells and α‐SMA+ epithelial cell area in the tongue and esophagus of the mice. Scale bars = 200 µm, 20 µm (inset). E,F) qRT‐PCR for the expression of E) proliferation marker genes (*Pcna*, *Ccnb1*, *Ccnd1*) and F) EMT‐related genes (*Cdh1*, *Cdh2*, *Snai1*, *Zeb1*, *Acta2*, *Twist1*, *Vim*) in the tongue of CTL, NA, and NA+BAPN mice. The data are expressed as mean ± s.e.m. (*n* = 3 or 6 mice per group). Statistical analysis involved one‐way ANOVA followed by post hoc Tukey's or Dunnett's test (*p < 0.05; **p  <  0.01; ***p  <  0.001; and ****p <  0.0001). Representative images are shown for H&E and IHC.

To assess the impact of ECM softening on tongue/esophageal SCC progression, we examined tissue specimens from each mouse using H&E staining and pan keratin, an epithelial cell‐specific marker used to detect epithelial tumor cells (**Figure** **7**A,B). Histological scoring demonstrated that in the NA group, 83% (5/6) of mice exhibited invasive carcinomas in the tongue and/or esophagus (Table S6, Supporting Information). Surprisingly, BAPN treatment significantly inhibited tongue/esophageal SCC progression in the NA+BAPN group, with the majority of pathological lesions in the tongue and esophagus exhibiting severe dysplasia (carcinoma in situ) (Table S6, Supporting Information). Immunohistochemistry for the proliferation marker Ki67 and RT‐qPCR analysis of proliferation markers such as *Pcna*, Cyclin B1 (*Ccnb1*), and *Ccnd1*, showed reduced cell proliferation in the NA+BAPN group compared to the NA group (Figure 7C,E). Additionally, α‐SMA‐expressing cells were less abundant in both the tongue and esophageal tissues of the NA+BAPN group compared to the NA group (Figure 7D). Notably, the cell types expressing α‐SMA differed between tongue and esophageal tissues. In the esophageal tissues, dysplastic epithelial cells or tumor cells expressed α‐SMA, indicating that they had undergone EMT. In contrast, in the tongue, α‐SMA‐expressing cells were primarily stromal cells, possibly CAFs, involved in ECM remodeling and increased tumor stiffness.^[^ ^18b^ ^]^ Based on RT‐qPCR data for the epithelial marker E‐cadherin (*Cdh1*) and several mesenchymal markers including N‐cadherin (*Cdh2*), *Snai1*, *Zeb1*, *Acta2*, *Twist1*, and *Vim*, in tongue tissues (Figure 7F), we assumed that significantly more CAF‐like mesenchymal stromal cells accumulated in the tongues of NA‐treated mice and that tongue SCC tumor cells underwent partial EMT, as previously reported in OSCC.^[^ ^39^ ^]^ Taken together, these results indicate that ECM softening achieved through BAPN treatment disrupts GLI2‐mediated proliferation and EMT in tongue/esophageal SCC, suggesting that ECM softening could be a potential means to prevent the progression of tongue/esophageal SCC.

### 2.8 Evidence in Patient Samples: Nuclear GLI2‐Positivity Is Closely Associated with Collagen Accumulation in OSCC

**Figure 8:**
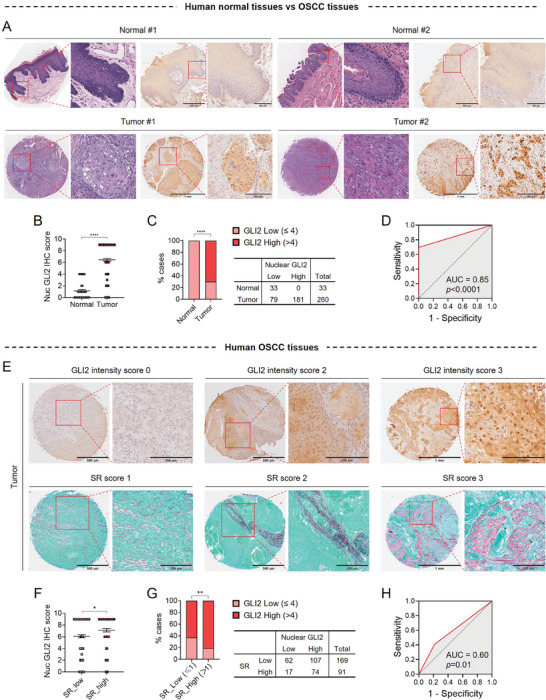
Nuclear GLI2 expression is closely associated with collagen accumulation in patients with OSCC. A) Representative images of H&E staining provided by TissueArray.Com. and IHC for GLI2 in tissue microarrays of human normal oral mucosal and OSCC tissues. B) The IHC scores for nuclear GLI2 expression, ranging from 0 to 9, for normal (*n* = 33) and tumor (*n* = 260) tissues. The mean mean ± s.e.m. results are displayed, and the Mann‐Whitney U test was conducted for statistics (****p <  0.0001). C) The proportions of tissue microarray cases grouped by low (≤ 4) or high (> 4) GLI2 expression in normal and tumor tissues. A table indicates the number of cases in each group. Chi‐square test was used for statistics (****p <  0.0001). D) ROC curves and the area under curve (AUC) value of high nuclear GLI2 expression for distinguishing OSCC tumors from normal tissues. E) Representative images of IHC for GLI2 or SR staining in serial sections of tissue microarrays of human OSCC tissues, showcasing varying GLI2 intensity scores (ranging from 0 to 3) and SR scores (ranging from 1 to 3). F) The IHC scores for nuclear GLI2 (ranging from 0 to 9) for OSCC tumor tissues with low SR scores (SR_low, ≤ 1) or high SR scores (SR_high, > 1). The mean ± s.e.m. results are displayed, and the Mann‐Whitney U test was performed (*p <  0.05). G) The proportions of tissue microarray cases grouped based on low (≤ 4) or high (> 4) GLI2 expression in cases with low SR scores (SR_low, ≤ 1) or high SR scores (SR_high, > 1). A table indicates the number of cases in each group. Chi‐square test was conducted (**p = 0.003). (H) The AUC of the ROC of high SR score in distinguishing OSCC tumors with high GLI2 expression from tumors with low GLI2 expression. Scale bars indicate the specified length (µm) under the bars.

Finally, we sought to elucidate the clinical relevance of stromal remodeling that increases tumor stiffness and its role in promoting tumor progression through the mechanical activation of GLI2. To enable this, we performed immunohistochemistry for GLI2 in tissue specimens obtained from a cohort of OSCC patients (*n* = 260) with heterogeneous collagen architecture. Normal tissue specimens (*n* = 33) were also included for comparative analysis. GLI2 expression was detected in 87.3% (227/260) of the OSCC tumor lesions and 51.5% (17/33) of the normal tissue lesions (**Figure** **8**A,B). The nuclear GLI2 score (ranging from 0 to 9), determined by combining the intensity score (ranging from 0 to 3) and the percentage score (ranging from 0 to 3), was significantly higher in tumors than in normal tissues (Figure 8A,B), indicating that GLI2 was hyperactivated in OSCC. We observed that all tissue cases exhibiting high GLI2 expression (score > 4) were of the tumor group (Figure 8C), and its Area Under the Receiver Operating Characteristic (AUROC) curve value for GLI2 in distinguishing tumor tissues from normal tissues was 0.85 (*p*  < 0.0001, Figure 8D). Notably, strong GLI2 expression (score ≥ 7) in the nuclei was observed in the majority of OSCC cases (58.1%, 151/260), but not in normal tissues. Tumor stiffness correlates with the accumulation of thick, densely packed collagen fibers surrounding tumor islands.^[^ ^6^ ^]^ Based on Sirius Red staining, we divided the OSCC specimens into two groups according to the Sirius Red score (ranging from 0 to 3): low (score ≤ 1) and high (score > 1) collagen fiber accumulation surrounding tumor lesions (Figure 8E). Of note, we observed higher nuclear GLI2 expression in OSCC tumor lesions with high collagen fiber accumulation with the AUROC value of 0.6 (*p* <  0.01) (Figure 8E–H), indicating a positive correlation between nuclear GLI2 positivity and collagen accumulation (indirectly reflecting tumor stiffness). These findings suggest that the stiffened tumor stroma may induce hyperactivation of GLI2 in tumor lesions, thereby promoting invasive growth of OSCC.

## 3 Conclusion

**Figure 9:**
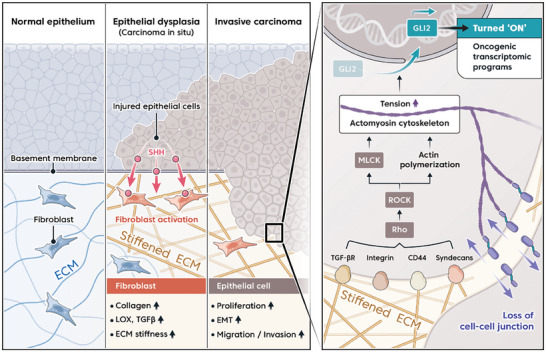
Schematic diagram of the mechanism by which stiffened matrices promote carcinogenesis through non‐canonical Hedgehog signaling. The diagram highlights the key events and signaling interactions that contribute to the transition from premalignant lesions in mucosal tissues to malignant carcinoma. Injured epithelial cells release Sonic Hedgehog (SHH), which is a ligand of the Hh signaling pathway. SHH promotes the activation of myofibroblasts, which in turn produce collagen and collagen‐remodeling enzymes like LOX, resulting in increased extracellular matrix (ECM) stiffness. Basal epithelial cells sense the increased ECM stiffness and enhance the formation of cell‐ECM adhesions mediated by collagen receptors such as CD44, Syndecans, and integrins. These cell–ECM adhesions are closely associated with actin polymerization and actomyosin cytoskeletal contractility. Myosin light chain kinase (MLCK) is involved in the regulation of MLC phosphorylation, contributing to actomyosin contractility. Importantly, these processes facilitate the nuclear translocation of GLI2, an oncogenic transcription factor, in response to the stiffened matrix. Within the nucleus, GLI2 orchestrates transcriptomic programs that drive cancer‐like transformations in epithelial cells, including enhanced cell proliferation and epithelial‐to‐mesenchymal transition (EMT). Consequently, epithelial cells lose cell‐cell junctions and acquire migratory and invasive properties, promoting the growth of invasive carcinoma. The diagrams provide a comprehensive overview of the complex interactions and molecular events in the progression of carcinogenesis, shedding light on the role of matrix stiffness and non‐canonical Hh signaling in this process.

Growing evidence suggests that elevated ECM stiffness not only results from but also fosters cancer development, progression, and metastasis.^[^ ^40^ ^]^ ECM stiffening is implicated in diverse cellular responses, exerting significant influence on biological processes such as proliferation, malignant transformation, and drug resistance.^[^ ^41^ ^]^ Consequently, cancer pathogenesis is mediated through a synergistic interplay between mechanical cues from the ECM and oncogenic signaling.^[^ ^42^ ^]^ Here we demonstrated that injured epithelial cells release SHH, which activates fibroblasts to produce collagen and collagen‐remodeling enzymes like LOX. Simultaneously, basal epithelial cells sense the increased ECM stiffness and undergo cancer‐like transformations, orchestrated by the transcriptomic programs of oncogenic GLI2 (as illustrated in **Figure** **9**). Importantly, we provided direct in vivo evidence of this process through mouse models of oral/esophageal injury and precisely engineered hydrogels that faithfully mimic the pathophysiological ECM stiffness. While our investigation is centered on tongue/esophageal SCC, the mechanism elucidated herein holds potential relevance to other progressive cancer types as well.

Utilizing recently advanced transcriptomics analysis tools, such as 10 × Genomics Visium and snRNA‐seq, this study revealed active interactions between epithelial cells and fibroblasts in a precancerous condition. These interactions involved the exchange of mechanoresponsive signals, such as collagen‐integrin signaling (Figure 4E–H, Figures S16 and S18, Supporting Information). The collagen‐binding integrin signaling initiates cellular mechanotransduction, leading to the formation of focal adhesions that harbor the ECM‐induced mechanical signalings and further transmit them along the actin bundles.^[^ ^43^ ^]^ While the integrins and focal adhesions jointly play a role in sensing the ECM stiffness, the dynamics of focal adhesions are intimately linked to actin polymerization and actomyosin contractility,^[^ ^43^, ^44^ ^]^ both of which are essential for the nuclear translocation of YAP in response to stiffened substrates.^[^ ^45^ ^]^ Our study, for the first time, suggests that, similar to YAP, GLI2 nuclear localization may also be influenced by actin polymerization and actomyosin contractility. This was evidenced by the observation that nuclear GLI2 expression decreased when cells were treated with LatA or Y27632, accompanied by a reduction in nuclear flatness (Figure 2C–E). Although the precise mechanism behind inhibition of GLI2 nuclear localization by tubacin‐mediated microtubule acetylation was not fully elucidated in this study, we observed a concurrent decrease in F‐actin intensity in oral epithelial cells upon tubacin treatment (Figure S4D, Supporting Information). This effect may be attributed to the decrease in HDAC6‐mediated cortactin deacetylation and subsequent actin polymerization.^[^ ^32a^ ^]^ Recent studies have explored the relationship between actomyosin contractility and microtubule mechanics, which can be influenced by microtubule polymerization,^[^ ^32^, ^46^ ^]^ and post‐translational modifications of α‐tubulin (e.g., acetylation).^[^ ^47^ ^]^ Given the emerging significance of the microtubules in cellular mechano‐responsiveness,^[^ ^48^ ^]^ investigating the role of microtubule mechanics in the context of oral/esophageal carcinogenesis is warranted in future studies.

While Hh signaling has shown promise as a therapeutic target due to its dysregulated activation in various cancers, its clinical application has been limited by resistance to anti‐Hh agents.^[^ ^49^ ^]^ Therefore, investigation of the underlying mechanisms responsible for cellular resistance to Hh inhibitors, particularly FDA‐approved Smo inhibitors like vismodegib and sonidegib for treating BCC,^[^ ^50^ ^]^ may provide valuable insights into the regulation of Hh signaling. The resistance to Smo inhibitors is attributed to various factors, including point mutations in the drug binding pocket or downstream molecules of Smo. Interestingly, recent research has shed light on non‐canonical mechanisms that strengthen Hh pathway output. For instance, non‐canonical activation of the Hh signaling pathway occurs through the transcriptional activation of Rho exchange factor and ARHGEF17 by AP‐1 and TGF‐β signaling.^[^ ^51^ ^]^ The activation of ARHGEF17 induces RhoA‐mediated actin polymerization, leading to nuclear localization of myocardin‐related transcription factor (MRTF) and possibly GLI2 itself. In the nucleus, MRTF acts as a positive transcription cofactor for GLI1, sharing chromosomal occupancy with GLI1, thus amplifying GLI1 transcriptional activity in resistant BCC.^[^ ^52^ ^]^ Additionally, the loss of primary cilia confers protection to tumor cells against Smo inhibitors, as it sustains GLI2 activity in resistant medulloblastoma cells.^[^ ^53^ ^]^ Our findings indicate that OSCC cells lack primary cilia, which diminish with increasing substrate stiffness (Figure S3, Supporting Information). Interestingly, reinforcing primary cilia through tubacin treatment inhibits GLI2 nuclear localization (Figure S4B,C, Supporting Information). These results suggest that ECM softening might be a viable strategy to reset the Hh signaling by targeting the non‐canonical Hh pathways, specifically suppressing TGF‐β signaling, which becomes hyperactivated in stiff ECM^[^ ^54^ ^]^ (Figure 4E–H; Figures S16 and S17, Supporting Information), and inducing the recovery of primary cilia. These insights will pave the way for potential therapeutic interventions to overcome drug resistance and enhance the effectiveness of Hh pathway‐targeted therapies in cancer treatment.

While this study primarily focused on the interaction between epithelial cells and the ECM, it is important to note that other cell types within the tumor microenvironment, including immune cells and endothelial cells, are also influenced by ECM mechanics, thereby impacting cancer progression.^[^ ^55^ ^]^ Specifically, T cells face physical exclusion from tumors due to the presence of fibrillar collagen‐rich ECM,^[^ ^56^ ^]^ and their effector function is compromised in stiff matrices,^[^ ^57^ ^]^ leading to immunosuppression and reduced effectiveness of immunotherapy. Additionally, impeded T cells may experience prolonged exposure to immune‐suppressive cytokines. To address these challenges, ECM softening has emerged as a potential strategy to enhance T cell migration and infiltration into primary and metastatic tumors, thereby improving the efficacy of immunotherapies like immune checkpoint inhibitors.^[^ ^58^ ^]^ ECM softening can be achieved through the inhibition of enzymes like LOX,^[^ ^6^ ^]^ as in our study or cytokines such as TGF‐β.^[^ ^57a^ ^]^ Moreover, T cells themselves can be engineered to secrete enzymes that facilitate ECM softening, thereby promoting tumor infiltration.^[^ ^59^ ^]^ Consequently, direct approaches involving ECM degradation as well as indirect methods through T cell engineering can be promising next‐generation immunotherapies by manipulating ECM mechanics. Future studies involving the characterization of T cells in our mouse models of tongue/esophageal SCC before and after BAPN treatment, along with evaluating the effectiveness of combining immune checkpoint inhibitors with ECM softening, hold significant value. These future studies have the potential to deepen our understanding of the intricate interactions between T cells and ECM mechanics and may contribute to advancing novel strategies for the treatment of tongue/esophageal SCC, and potentially other cancers.

Finally, our findings demonstrated the mechanisms involved in the progression from premalignant lesions in mucosal tissues to malignant SCC, highlighting the key early events and signaling interactions that drive malignancy. These events include the evolution of SHH‐induced CAFs, the ECM stiffening driven by myofibroblastic CAFs, and the stiffness‐responsive, GLI2‐dependent reprogramming of premalignant epithelial cells. In line with our findings, Chen et al.^[^ ^60^ ^]^ have recently conducted a comprehensive study on esophageal SCC, demonstrating altered transcriptional landscape and ligand‐receptor interactions between epithelial cells and fibroblasts as early as the high‐grade intraepithelial neoplasia stage, contributing to the development of malignancy. In contrast to focusing solely on interactions mediated by chemical factors, our study emphasizes epithelial‐stromal interactions mediated by altered matrix mechanics resulting from activated fibroblasts. Specifically, we concentrate on actin‐mediated mechanotransduction in survived epithelial cells which are exposed to elevated stiffness, leading to the physical activation of oncogenic transcription factors. Both studies converge on the notion that epithelial‐stromal interactions, culminating in the formation of tumor‐promoting CAFs, play a pivotal role in epithelial malignant transformation, preceding the establishment of a T cell‐excluded or immunosuppressive tumor microenvironment.^[^ ^21b^ ^]^

Specifically, this study proposes that stiffness measurement of oral cavity and esophageal tissues offers a promising approach for the early detection of individuals at high risk of developing SCC. Recent works have explored noninvasive methods, such as aspiration‐based techniques^[^ ^61^ ^]^ and strain elastography combined with imaging,^[^ ^62^ ^]^ to assess the relative stiffness of precancerous and cancerous tongue tissues. By evaluating tissue stiffness, clinicians may be able to identify patients in the early stages of the disease, enabling timely interventions and improving patient outcomes. In summary, the current study emphasizes the importance of considering the mechanical properties of the precancerous and cancerous microenvironments, as perceived by cells, in order to understand the mechanobiological dialogues between cells and the ECM. Furthermore, our findings provide valuable insights into the role of ECM stiffness in driving oral/esophageal carcinogenesis, which can be leveraged for diagnostic purposes and to re‐strategize the targeted drug therapies in oral/esophageal SCC.

## 4 Experimental Section

### Drugs and Chemicals

Smoothened Agonist HCl (SAG, S7779, Selleckchem.com, 2 × 10^−6^ m), Sonic Hedgehog human (SHH, SRP3156, Sigma, 2 × 10^−6^ m), Vismodegib (GDC‐0449, S1082, Universal Biologicals, 20 × 10^−6^ m), transforming growth factor β (TGF‐β, 100‐36E, Peprotech, 10 ng mL^−1^), GANT61 (G9048, Sigma, 15 × 10^−6^ m), Y‐27632 (#1254, TOCRIS, 40 × 10^−6^ m), Latrunculin A (LatA, #3973, TOCRIS, 0.2 × 10^−6^ m), and Tubacin (Tub, #3402, TOCRIS, 5 × 10^−6^ m) were used for in vitro experiments.

4‐Nitroquinoline N‐oxide (4‐NQO, N8141, Sigma, 200 µg mL^−1^) and Arecoline hydrobromide (A0523, TCI, 500 µg mL^−1^) were dissolved in drinking water to induce tongue/esophageal injury in mice throughout the experimental period. SAG (25 mg kg^−1^ day^−1^ twice a week; O.G.), β‐Aminopropionitrile fumarate salt (BAPN, A3134, Sigma, 100 mg kg^−1^ day^−1^ twice a week; I.P.), and D‐Sorbitol (S1876, Sigma, 30 µg mL^−1^) were also used in this study.

### Human Subjects

The pathological tissue lesion was obtained post‐surgically from a patient diagnosed with leukoplakia at the Department of Oral and Maxillofacial Surgery of Dankook University Dental Hospital (Cheonan, Republic of Korea) and immediately processed for spatial transcriptomics and snRNA‐seq analyses. For the comparison, normal gingival tissue was obtained from the patients who visited Dankook University Dental Hospital for third molar extractions. The use of human tissue was approved by the Institutional Review Board of Dankook University Hospital (DKUDH IRB 2021‐7‐004). All patient identifications were removed, and the specimens were renumbered for the record. Human samples were used in accordance with the National Institutes of Health and institutional guidelines for human subject research.

### Spatial Transcriptomics (10× Genomics Visium)

Fresh tissues were embedded in optimal cutting temperature (OCT) compound in cryomolds (15 × 15 mm) over the dry ice and then stored at −80 °C. The frozen tissue was sectioned into 10 µm thickness with a cryostat (CryoStar NX70, ThermoFisher Scientific) and then fixed in pre‐chilled methanol for 30 min at −20 °C. Hematoxylin and eosin (H&E) staining was carried out to examine the histology and determine the specific area (6.5 × 6.5 mm) for sequencing. Imaging was performed using a Nikon Eclipse Ti2 microscope with a 10x lens magnification. The tissue section was placed on a Visium array slide (Visium Spatial Gene Expression slide, 10 × Genomics, https://10xgenomics.com/) and processed according to the Visium Spatial Gene Expression User Guide (10 × Genomics). This involved permeabilization of the section for 15 min, followed by on‐slide reverse transcription (RT) to generate complementary DNA (cDNA) libraries. The quality of the cDNA libraries was assessed, and sequencing was performed using an Illumina NovaSeq 6000 system. The sequencing depth achieved was approximately 52K reads per spot. Data analysis was conducted using the Space Ranger (v1.1) pipelines provided by 10 × Genomics. The raw data was processed with the mkfastq pipeline to demultiplex the samples into FASTQ files. The count pipeline was then used for alignment, barcode/unique molecular identifier (UMI) counting, and read mapping to the human reference transcriptome (GRCh38), with the aid of bright‐field microscope images. Further analysis of the data was performed using the Seurat package (v4.1.0) in R (v4.2.1). The data was normalized using SCTransform (v0.3.3), and principal component analysis (PCA) and uniform manifold approximation and projection (UMAP) dimensionality reduction were conducted with the first 30 PCs. Clustering was performed using the FindClusters function in Seurat with a resolution parameter of 1.0. The cell types of each cluster were inferred and refined by overlaying the clusters onto the associated histology images and identifying marker genes using the FindAllMarkers function. Spatial feature expression plots were generated using the SpatialFeaturePlot function in Seurat. To gain insights into the functional characteristics of the identified cell populations, Gene Ontology (GO) enrichment and Kyoto Encyclopedia of Genes and Genomes (KEGG) pathway analyses were performed using the gprofiler2 (v0.2.1) R package. Significant enrichment of GO terms and pathways was determined based on an adjusted *p*‐value threshold of less than 0.05. Expression scores for specific gene sets were calculated using the AddModuleScores function in Seurat with default parameters. Cell–cell interaction analysis was conducted using the CellChat pipeline with default options.

### Single‐Nucleus RNA Sequencing (snRNA‐Seq)

The tissues were mechanically homogenized in lysis buffer containing of 10 × 10^−3^ m Tris‐HCl, 10 × 10^−3^ m NaCl, 3 × 10^−3^ m MgCl2, and 0.025% Lysis Reagent. The resulting nuclear suspension was centrifuged at 500 × *g* for 10 min at 4 °C and the pellet was resuspended in 1 mL wash buffer consisting of 1 × phosphate‐buffered saline (PBS), 1% bovine serum albumin (BSA), and 0.2 U µL^−1^ RNase inhibitor. The nuclear suspension was filtered twice using 40 µm Flowmi cell strainer and then centrifuged at 500 × *g* for 10 min at 4 °C. The resulting pellets were then resuspended in wash buffer on ice and immediately processed with the Chromium Next Gem Single Cell 3′ v3.1 protocol from 10 × Genomics. Libraries for snRNA‐seq were generated and sequenced on an Illumina NovaSeq 5000/6000 S2 with NovaSeq 6000 system, with a sequencing depth of approximately 41–83 K reads per nucleus. The sequencing data was aligned to the reference human genome (GRCh38) using CellRanger (v6.1.2) from 10 × Genomics. The output data was processed using R (v4.2.1) and Seurat (v4.1.0). Nuclei with fewer than 200 unique genes, more than 1 million UMIs, predicted doublets and/or > 20% mitochondrial reads were filtered out. Potential doublets were detected and removed using doubletFinder. The expression data was then normalized using SCTransform (v0.3.3). Integration of datasets was performed using FindIntegrationAnchors and IntegrateData, with 30 dimensions. PCA was performed using RunUMAP, and the data was visualized using Seurat. Data scaling, PCA, selection of PCs, clustering and visualization were proceeded as described above at a resolution 0.1 with 30 PCs. Cluster annotations were assigned using the FindAllMarkers function with a logistic regression test and genes were selected as markers based on Bonferroni‐adjusted *p*‐value <  0.05. Cell types were assigned based on manual curation of known marker genes from PanglaoDB. Cell‐Cell interactions were analyzed using the CellChat pipeline with default options. To compare gene expression between normal and leukoplakia samples, the Mann‐Whitney U test was performed to obtain a mean ranks and standard errors of mean (s.e.m.) for each gene. The proportion of positive nuclei per cluster in each group was calculated and visualized using pie charts and bar plots. Differential gene expression between GLI2‐positive and GLI2‐negative nuclei was analyzed using a threshold of log_2_ fold change (FC)> 0 and *p*‐value <  0.05. GO enrichment and KEGG pathway analyses were performed using the gprofiler2 package (v0.2.1), considering GO terms and pathways significantly enriched at an adjusted *p*‐value less than 0.05. GraphPad Prism 8 and R were used for data visualization.

### Animal Studies

Forty‐one 6‐week‐old, male C57BL/6 mice (weighing 20–22 g) were purchased from JSBIO (Republic of Korea) and housed in a specific pathogen‐free animal facility under controlled temperature (22 ± 2 °C), humidity (55 ± 5%), and photoperiod (12‐h light/dark cycle). For NA±SAG model of tongue/esophageal epithelial dysplasia, mice (*n* = 20) were fed with standard diet and water dissolved with NA (4‐NQO + Arecoline), together with D‐Sorbitol to prevent the bitter taste, ad libitum for 8 weeks to induce tongue/esophageal injury. On the 5^th^ week of NA treatment, a half of the mice were additionally fed with SAG until the end of the treatment period to determine the effect of Hh signaling in progression of tongue/esophageal injury. To develop tongue/esophageal SCC in mice (NA±BAPN model of tongue/esophageal SCC), mice (*n* = 12) were exposed to NA‐dissolved water for 16 weeks (exposure period) and then NA‐removed plain water for 10 weeks (development period). A half of mice were received BAPN for exposure period from the 8^th^ week of NA treatment to examine anti‐cancer effect of ECM softening by LOX inhibition. As a control group, age and sex‐matched mice (*n* = 3 or 6) fed with standard diet and plain water throughout the experimental period were used. SAG and BAPN were given twice a week with the dosage and method as mentioned above. At the end of the experimental period, the mice were fasted overnight and euthanized by CO_2_ asphyxiation for 2 min in a euthanasia apparatus followed by blood collection from the abdominal vein. The coagulated blood samples were centrifuged at 3000 × *g* for 15 min at 4 °C and the serum was stored at −80 °C until further analysis. Finally, tongue and esophagus were dissected, washed with PBS, weighed, and fixed for histological analysis or snap‐frozen and then stored at −80 °C until future analysis.

All experimental protocols were approved by the Dankook University (DKU‐23‐014, DKU‐23‐015) Institutional Animal Care and Use Committee (IACUC) as per the National Research Council “Guide for the Care and Use of Laboratory Animals”.

### Tissue Stiffness Measurement

Fresh tongue tissues dissected from the control, NA‐, and NA+SAG‐treated mice were longitudinally cut, embedded in OCT compound, and immediately stored at −80 °C. Frozen tissues were sectioned into 20 µm thickness serially and adjacent sections were used for either Sirius Red collagen staining or stiffness measurement. For nanoindentation, cryosections were collected on a 25 mm silanized cover glass and washed with HPBS containing calcium. During the measurement specimens were immersed in HPBS solution. Nanoindentation was conducted using Pavone (Optics11 Life nanoindenter, Netherlands) according to the manufacturer's instructions. Ten spots in staining‐proven collagen‐rich ECM were randomly selected per group for evaluation.

### Histology and Immunohistochemistry (IHC)

Tissues were fixed immediately after collection in a 10% neutral buffered formalin solution, embedded in paraffin (SAKURA Tissue‐Tek TEC 5 Tissue Embedding Center, Japan), and cut into 5 µm sections (Leica RM 2135 microtome, Germany). For histological analysis, tissue sections were deparaffinized, hydrated, and subjected to H&E and Sirius Red/Fast Green collagen staining (Picro‐Sirius Red Solution, ab246832, abcam; Fast Green FCF, F7252‐5G, Sigma, 0.1%). For IHC, deparaffinized and rehydrated tissue sections were incubated in 3% hydrogen peroxide dissolved in methanol to quench endogenous peroxidase. Antigen retrieval was performed by heating in 10 × 10^−3^ m citrate buffer (pH 6.0) for 10–15 min using a microwave. After cooling to room temperature, tissue sections were washed with tris‐buffered saline for 5 min and nonspecific antibody‐binding was prevented using serum‐free Protein Block solution (X090930‐2, Agilent, Dako) for 30 min. The sections were then probed with primary antibodies overnight at 4 °C and the antibodies used in this study are as follows; anti‐SHH (ab53281, 1:200, abcam), anti‐YAP (#14074, 1:200, Cell signaling), anti‐GLI2 (18989‐1‐AP, 1:200, Proteintech), anti‐Ki67 (ab15580,1:200, abcam), anti‐α‐Smooth Muscle Actin (#19245, 1:200, Cell signaling), anti‐Pan‐Keratin (#67306, 1:200, Cell signaling), anti‐Vimentin (#5741, 1:200, Cell signaling), and anti‐E‐cadherin (#14472, 1:200, Cell signaling). Horseradish peroxidase (HRP)‐conjugated secondary antibody (EnVision+/HRP, Rabbit, K400311‐2, Agilent, Dako) was applied for 30 min and the proteins were visualized in brown using 3,3′‐diaminobenzidine (Liquid DAB+, K346811‐2, Agilent, Dako) and counterstained with hematoxylin.

### Tissue Microarray (TMA) Analysis

Normal and OSCC TMAs (OR208a, OR481a, OR601c) were purchased from TissueArray.Com (USA). Sirius Red staining and IHC for GLI2 were conducted for all three TMAs. Images were captured using a slide scanner (OlyVIA 3.2, VS2000 ASW, Olympus) and H&E‐stained images were provided by TissueArray.Com. A pathologist with specialized pathology training evaluated staining semi‐quantitatively as previously described.^[^ ^63^ ^]^ For GLI2 IHC, cells showing nuclear staining were regarded as positive. The intensity of staining (0, negative; 1, weak; 2, moderate; and 3, strong) and the percentage of positive cells (0, 0%; 1, 1%–10%; 2, 11%–50%; and 3, 51%–100%) were scored. Final IHC scores were established by multiplying the intensity and percentage scores. The final scores ≤4 were considered as low GLI2 expression, while those >4 were considered as high expression. Sirius Red staining was evaluated quantitatively as previously described with slight modifications.^[^ ^64^ ^]^ The proportion of Sirius Red‐positive area was calculated in the stroma surrounding tumor and scored as follows: 1, 0%–25%; 2, 26%–50%; and 3, 51–100%. Score 1 was considered as low collagen deposition, while scores 2 and 3 were considered as high collagen deposition.

### Evaluation of Mouse Tissue Staining

The stained mouse tissue sections were imaged using a slide scanner (OlyVIA 3.2, VS2000 ASW, Olympus). Sirius Red‐positive area was analyzed in the stroma underneath epithelium or surrounding tumor using Image J 1.8.0 software (http://imagej.nih.gov/ij/index.html) and the relative collagen‐deposited area was calculated in comparison to the control group. To evaluate nuclear YAP+, GLI2+, or Ki67+ epithelial cells, three or five different fields of same square area of epithelium for each mouse were randomly selected and the proportion (%) of nuclei with positive signal to total nuclei were calculated. Similarly, cytoplasmic SHH+ epithelial cell proportions (%) to total epithelial cells were measured using Image J 1.8.0 software. α‐SMA+ area (%) was evaluated in epithelial or tumor area of randomly selected three different fields for each mouse. Measurements of tongue and esophageal epithelium thickness were performed using three representative microscopic fields of H&E‐stained images for each mouse. The lowest and the highest thickness of the epithelium was measured using Image J 1.8.0 software and then the average epithelium thickness was calculated for each individual. To assign a histological grading, H&E and Pan‐Keratin immunohistochemically stained sections were carefully evaluated for each mouse. The histologically nontumorous tongue and esophageal tissue regions were considered as internal negative control for each mouse. Under a low (4 × lens) or a high (20 × lens)‐power field, all the regions of each slide were evaluated independently by two investigators in a blinded fashion. The histological state of each section was scored as follows: 0, equivalent to the negative control; 1, hyperplasia; 2, mild dysplasia; 3, moderate dysplasia; 4, severe dysplasia; and 5, invasive carcinoma. The contribution (%) from 0 to 100% was assigned for each histological state based on the scoring.

### Synthesis of Methacrylated Hyaluronic Acid (MeHA) Hydrogel

The HyActive powder (a low molecular sodium hyaluronate) was purchased from Contipro Inc. (ProTec Ingredia Ltd, Borehamwood, UK). The thiolated RGD‐containing peptide with a designed sequence of GCGYGRGDSPG (C40H60N14O16S1, MW 1 KDa, 96% HPLC purity) was supplied by Peptron (Daejeon, Republic of Korea). The synthesis of MeHA was performed by direct immobilization of the methacrylate group of methacrylic anhydride (64100, Sigma) to the hydroxyl group of HA backbone by following previously published report.^[^ ^65^ ^]^ Briefly, sodium salt of hyaluronic acid (NaHA, MW 25–45 kDa) was dissolved with a 2% (w/v) concentration in distillated water overnight. Then, methacrylic anhydride (5.6 mL) was added to the previous solution and the reaction was continued for 24 h. During the reaction, the reaction temperature was maintained constant at around 0 °C with aid of an ice bath. Moreover, the solution pH was continuously monitored and controlled to fall in the range of 8 to 9 by drop‐wise addition of sodium hydroxide (5 m, S0899, Sigma) using a semi‐automatic syringe‐pump setup. Then, the obtained solution was filtered through Steritop and dialyzed via dialysis tubing (MWCO 12–14 kDa) against 5 L of distillated water at 4 °C, changing the water 3 times per day for 7 days. In the end, the polymer solution was again filtered, frozen down to −80 °C, and freeze‐dried at −40 °C for the next 7 days. The achieved white cottony material was stored at −20 °C before use. MeHA hydrogels with different surface stiffnesses were prepared by photo‐polymerization of MeHA solution. To this end, for both soft and stiff platforms, MeHA with 3% (w/v) was dissolved in a Tris‐HCl buffer containing 1 mg mL^‐1^ of LAP photoinitiator. Thiolated RGD‐containing peptide (RGD‐SH) was also incorporated with 1 × 10^−3^ m concentration to MeHA for 2 h at room temperature to provide sufficient cell adhesion property. To keep the resulting platform thin enough for better imaging, stay submerged in media, and linger hydrated, a 100 µm layer of MeHA gel was prepared by photocrosslinking 19 µL of MeHA solution sandwiched between two cover glasses (a bottom methacrylated coverslip and a top untreated 15 mm coverslip). Soft and stiff platforms were created by applying 20 and 60‐second UVA (OmniCure 320–390 nm wavelength) with 350 µW cm‐^2^, respectively.

### Cell Culture

Human gingival fibroblast (HGF), human dermal fibroblast (HDF), immortalized human oral keratinocytes (iHOK), and several OSCC cell lines (HSC2, HSC3, MC3, HN22, and Ca 9.22) were used in this study. Cells were maintained at 37 °C and 5% CO_2_ in a specific culture medium as follows: HGF, minimum essential media alpha modification (Alpha MEM, LM 008‐53, WELGENE); HDF, Dulbecco's modified Eagle's medium (DMEM, high glucose, LM 001‐05, WELGENE); iHOK, GM−2 Keratinocyte Growth Medium‐2 BulletKit (KBM‐2, CC‐3107, Lonza); HSC2, RPMI1640 (LM 011‐03, WELGENE); and other OSCC cell line (HSC3, MC3, HN22, and Ca 9.22), DMEM/F‐12 (11320033, Gibco) together with 10% fetal bovine serum (FBS, 35‐015‐CV, Corning) and 1% penicillin‐streptomycin (PS, 15140163, Gibco).

### Primary Oral Epithelial Cell Isolation

Normal gingival tissues were obtained from patients undergoing third molar extractions at Dankook University Dental Hospital. The tissue specimens were transported to the cell culture laboratory in MACS Tissue Storage Solution (130‐100‐008, Miltenyl Biotec,) and the epithelial tissue layer was carefully separated from the connective tissue using a sterilized surgical knife and forceps. Subsequently, the epithelial tissues were incubated in a solution containing 3% penicillin and streptomycin for 30 min at room temperature to prevent microbial growth. The tissues were then placed in a cell culture dish, allowed to dry, and firmly attached to the culture plate. Next, the tissues were flooded with keratinocyte growth medium (KGM−2 Keratinocyte Growth Medium‐2 BulletKit, CC‐3107, LONZA) containing 10% FBS. The culture plate was then incubated at 37 °C in a humidified atmosphere of 95% air and 5% CO_2_. The culture medium was replaced with fresh medium twice a week. Once the squamous‐shaped epithelial cells began to proliferate around the tissue sample and reached approximately 50% confluence, the oral mucosal epithelial cells were harvested using a Trypsin‐EDTA (0.25%) solution (25200 056, Gibco) at 37 °C. The disaggregated cells were collected, counted, and used for further investigations.

### SMO Knockdown Assay

To generate a cell line with SMO deletion, HSC3 cells were plated and infected SMO/smoothened shRNA (h) lentiviral particles (sc‐75329‐V, Santa Cruz) or control shRNA lentiviral particles (sc‐108080, Santa Cruz) at a multiplicity of infection (MOI) of 2.25, along with 5 µg mL^−1^ polybrene (sc‐134220, Santa Cruz), following the manufacturer's protocol.

(1) $$\mathrm{MOI}\;=\frac{\mathrm{Transduction}\;\mathrm{unit}\;\left(\mathrm{TU}\right)\;\mathrm{of}\;\mathrm{lentiviral}\;\mathrm{particles}\;}{\mathrm{Total}\;\mathrm{number}\;\mathrm{of}\;\mathrm{cells}\;\mathrm{per}\;\mathrm{well}}\;$$

After 24 h, the culture medium containing lentiviral particles was replaced with complete optimal medium without polybrene. To select stable clones expressing the shRNA, cells were split at a ratio of 1:5 and incubated for 48 h in complete medium, followed by treatment with puromycin dihydrochloride (sc‐108071, Santa Cruz) at a concentration of 10 µg mL^−1^ to eliminate non‐transduced cells. Successful transduction was confirmed by qRT‐PCR.

### Cytotoxicity

Cellular toxicities of inhibitors (tubacin, GANT61, Y27632, and LatA) were monitored with Cell Counting Kit‐8 (CCK‐8, CK04‐20, Dojindo Molecular Technologies). Briefly, cells were seeded at a density of approximately 5000 cells per well in 96‐well plates. After 24 h, cells were treated with the serial concentrations of inhibitors for 24 h and then the culture medium was replaced with 10% (v/v) CCK‐8 reagent diluted in the culture medium. After 2 h of incubation, the absorbance at 450 nm was measured using a Varioskan LUX multimode microplate reader (3020‐80732, ThermoFisher Scientific). The cell viability (%) was calculated using the following equation:

(2) $$\mathrm{Cell}\;\mathrm{viability}\;\%\;=\;100*\;\left(\frac{X_{1}-{\bar{X}}_{0}}{{\bar{X}}_{\mathrm{Ctrl}}-\;{\bar{X}}_{0}}\right)$$

where $X_{1},\;{\bar{X}}_{0}$, and ${\bar{X}}_{\mathrm{Ctrl}}$ refer to the average OD value of experimental (inhibitor‐treated cells) wells, of blank (no cells) well, and of control (vehicle‐treated cells) wells, respectively.

### Cell Immunofluorescence Staining

The cultured cells were fixed with 4% paraformaldehyde for 15 min at room temperature and permeabilized for 5 min at room temperature using 0.1% Triton X‐100 in PBS. Subsequently, cells were incubated with 10% normal goat serum (50062Z, ThermoFisher Scientific) for 1 h at room temperature to block nonspecific bindings. For staining, cells were incubated at 4 °C overnight with primary antibodies as follows: anti‐YAP (D8H1X) (1:100, 14074, Cell Signaling), anti‐GLI2 (18989‐1‐AP, 1:200, Proteintech), anti‐Acetylated‐tubulin (1:1000, T6793, Sigma), and anti‐HDAC6 (12834‐1‐AP, 1:200, Proteintech) diluted in 2% normal goat serum. The corresponding secondary antibodies, either Fluorescein (FITC) AffiniPure Donkey Anti‐Mouse IgG (H + L) (715‐095‐150, Jackson ImmunoResearch Laboratories), Rhodamine (TRITC) AffiniPure Donkey Anti‐Mouse IgG (H+L) (715‐025‐150, Jackson ImmunoResearch Laboratories), Fluorescein (FITC) AffiniPure Donkey Anti‐Rabbit IgG (H+L) (711‐095‐152, Jackson ImmunoResearch Laboratories), or Rhodamine (TRITC) AffiniPure Donkey Anti‐Rabbit IgG (H+L) (711‐025‐152, Jackson ImmunoResearch Laboratories) diluted in 2% normal goat serum, were used for the visualization. For F‐actin and nuclei staining, cells were treated with Alexa Fluor 546/488 Phalloidin (A22283/A12379, Invitrogen) and 4′,6‐diamidino‐2‐phenylindole, dihydrochloride (DAPI, D1306, ThermoFisher Scientific), respectively. Finally, cells were mounted using Fluoromount Aqueous Mounting Medium (F4680, Sigma).

### Microscopy and Image Analysis

The fluorescence images were acquired under an LSM700 confocal laser scanning microscope (Carl Zeiss, Oberkochen, Germany) using the Z‐stack function for all channels. To determine the nuclear to cytoplasmic ratios of YAP and GLI2, the mean fluorescence intensity in the nucleus was measured and divided by the mean fluorescence intensity in the cytoplasm. The cell area (i.e., 2D spreading area), perimeter, and aspect ratio of each cell were measured automatically using the Image J 1.8.0 software and the following formula was applied to calculate cell circularity:

(3) $$\mathrm{Circularity}\;=\frac{4*\pi *\left(\mathrm{cell}\;\mathrm{area}\right)}{{\left(\mathrm{Cell}\;\mathrm{perimeter}\right)}^{2}}\;$$

In this equation, values between 0.6–1 indicate higher cell roundness and values in the range 0–0.5 indicate elongated cells.

The percentage of ciliated cells was calculated by dividing the number of cells expressing primary cilia by the number of all cells per field, which was manually counted using Image J 1.8.0 software. Approximately 4 to 5 microscopic fields under 40 × or 63 × magnification were randomly examined from three to six repetitions for each experimental condition.

To analyze F‐actin intensity, 30 randomly selected phalloidin‐stained cells from 40× images for each experimental condition were examined using Image J 1.8.0 software. Equal numbers of images were taken in triplicate experiments.

The nuclear flattening index (NFI) was determined by analyzing DAPI‐stained images acquired with a Nikon Eclipse Ti2 microscope at a magnification of 60 ×. The NIS‐Elements AR software was utilized for image analysis, and the assays were performed in triplicate.

### Proliferation Assay

HSC3 cells were seeded on soft (5 kPa) or stiff (20 kPa) MeHA hydrogels with or without treatment of GANT61 (15 × 10^−6^ m) for 24 h. Afterward, cells were incubated with EdU‐containing solution (20 µM, Click‐iT EdU Cell Proliferation Kit, ThermoFisher Scienctific) for 8 h. After removing the media, cells were fixed with 4% formaldehyde in PBS for 15 min and washed twice with 3% BSA in PBS. After aspirating the BSA solution, cells were incubated with 0.5% Triton X‐100 for 20 min for permeabilization and washed twice with 3% BSA in PBS. Then, cells were incubated with 0.5 mL of Click‐iT reaction cocktail for 3 min at room temperature and washed twice. Nuclear staining was done with DAPI. Assays were repeated three times.

### Collective Migration Assay

HSC3 cells were cultured on soft (5 kPa) and stiff (20 kPa) MeHA hydrogels with or without treatment of GANT61 (15 × 10^−6^ m) for 24 h. A PDMS mold was used to confine cells on the center of hydrogels. Cells were rinsed with PBS which was then replaced with culture media containing 1% FBS and 10 µg mL^−1^ mitomycin C. GANT61 (15 × 10^−6^ m) treatment was kept in the group of GANT61‐treated cells. Collective cell migration was monitored using the JuLI Stage automated imaging system (NanoEntek Inc., Seoul, Republic of Korea). The migration speed was calculated using time‐lapse images taken at 20 × magnification every 20 min over 18 h. Assays were repeated four times.

### Transwell Migration Assay

HSC3 cells were seeded on tissue culture plate (TCP) and treated with GANT61 (15 × 10^−6^ m) for 24 h. Then, cells were detached from TCP and suspended in serum‐free media. 200 µL of serum‐free media containing approximately 5 ×  10^4^ cells were added to the insert of a Boyden chamber (Corning Inc., Corning, NY, USA) with 3 µm diameter pores. GANT61 (15 × 10^−6^ m) was kept being treated to GANT61‐treating group. 600 µL media containing serum as a chemoattractant was added inside the bottom well. After 48 h incubation, cells passed from the insert pores were fixed with 4% paraformaldehyde and stained with 0.1% crystal violet solution. Bright‐field images were taken with Olympus IX53P1F microscope under 20 × /0.45 ph1 lens and the number of migrated cells per 10 × field was quantified. Assays were repeated four times.

### Invasion Assay

HSC3 cells were seeded on TCP and treated with either GANT61 (15 × 10^−6^ m) or vehicle for 24 h. Then, cells were detached from TCP and suspended in serum‐free media. 200 µL of serum‐free media with approximately 5 × 10^4^ cells were added to the insert of a Boyden chamber (Corning Inc., Corning, NY, USA) with 3 µm diameter pores. Prior to the experiment, the inserts were coated with 50 µL of collagen I (1.2 mg mL^−1^) and allowed to dry overnight. 600 µL media containing serum was added inside the bottom well for chemoattraction. After 48 h incubation, cells passed from the insert pores were fixed with 4% paraformaldehyde and stained with 0.1% crystal violet solution. Bright‐field images were taken with Olympus IX53P1F microscope under 20 × / 0.45 ph1 lens and the number of migrated cells per 10 × field was quantified. Assays were repeated four times.

### Reverse Transcription (RT) Real‐Time Quantitative Polymerase Chain Reaction (qPCR)

Total RNA was extracted from tissues or cells using Ribospin II (GeneAll Biotechnology, Republic of Korea) according to the manufacturer's instructions. The concentration and purity of RNA were determined using NanoDrop 2000 (ThermoFisher Scientific) and template cDNA was synthesized using AccuPower Rocketscript Cycle RT Premix (BIONEER, Republic of Korea) according to the manufacturer's protocol. The SensiMix SYBR Hi‐ROX Kit (QT605‐05, Meridian Bioscience) was used for real‐time quantitative polymerase chain reaction (qPCR) on the manufacturer's specifications (StepOnePlus Real‐Time PCR System, Applied Biosystems). The qPCR results were normalized to the housekeeping gene GAPDH based on the threshold cycle (*C*_t_) and relative fold‐change was determined using the 2^−ΔΔCt^ method. The sequences of primers used in this study are listed in Table S7, Supporting Information.

### Enzyme‐Linked Immunosorbent Assay (ELISA)

The Sonic Hedgehog Human ELISA kit (ab100639, abcam) was used to measure serum level of SHH in mouse models, following the provided instructions by the manufacturer. Briefly, 100 µL of each standard and test sample were placed into the appropriate wells and incubated overnight at 4 °C with gentle shaking. Subsequently, biotinylated SHH detection antibody was added to each well and allowed to incubate for 1 h at room temperature with gentle shaking. Afterward, 1 × HRP‐streptavidin solution was added to each well and incubated for 45 min at room temperature. The TMB One‐Step substrate reagent was then added and incubated for 30 min at room temperature in the dark. Finally, stop solution was added to each well, and the absorbance was immediately measured at 450 nm. Data analysis and calculations were performed following the guidance provided by the manufacturer.

### Immunoblot

For the extraction of total proteins, cells were lysed in RIPA buffer (EBA‐1149, ELPISBIO) supplemented with Halt Protease and Phosphatase Inhibitor Cocktail (78444, ThermoFisher Scientific). The protein content in the lysates was quantified using the Pierce BCA Protein Assay Kit (23225, ThermoFisher Scientific). Equal amounts of total proteins (30 µg) were boiled with 5 × loading buffer at 100 °C for 5 min and separated by SDS‐PAGE on 8–15% protein gels. After separation, the proteins were transferred onto polyvinylidene difluoride membranes (PVDF, IPVH00010, Millipore). To prevent nonspecific binding, the membranes were blocked with 5% skim milk in TBS with 0.05% Tween‐20 (TBS‐T) for 2 h at room temperature. Subsequently, the membranes were incubated overnight at 4 °C with primary antibodies against α‐SMA (#19245, 1:1000, Cell signaling), LOX (ab174316, 1:1000, abcam), LOXL2 (NBP2‐75559, 1:1000, Novus Biologicals), GLI2 (NB600‐874, 1:1000, Novus Biologicals), RhoA (ab187027, 1:1000, abcam), ROCK (ab45171, 1:1000, abcam), p‐MLC (#3671, 1:1000, Cell signaling), MLC (#8505, 1:1000, Cell signaling), and GAPDH (ab110305, 1:1000, abcam). Following primary incubation, the membranes were exposed to the corresponding HRP‐conjugated secondary antibodies at room temperature for 2 h. Protein bands were visualized using the SuperSignal West Pico PLUS Chemiluminescent Substrate (34580, ThermoFisher Scientific) and imaged with the iBright 1500 (Invitrogen). Densitometric analysis for protein semi‐quantification was performed using Image J 1.8.0 software, with the band density of each target protein normalized to the density of its respective loading control (GAPDH).

The Active Rho Detection Kit (#8820, Cell Signaling) was used to detect the active form of RhoA, in combination with an immunoblot assay following the manufacturer's protocol. Briefly, the cells were harvested and washed with ice‐cold PBS, and then lysed in RIPA buffer. The cell lysates were centrifuged at 16 000 × *g* for 15 min to remove cell debris. Agarose beads were resuspended in glutathione resin, added to the spin cup with collection tube and mixed with GST‐Rhotekin‐RBD. The cell lysate was immediately transferred to the spin cup and the mixture was vortexed and incubated at 4 °C for 1 h with gentle rocking to allow binding of active RhoA to the resin. The spin cup with the bound activated RhoA was then centrifuged at 6000 × *g* for 30 s to separate the beads from the rest of the solution. After washing steps, the samples were mixed with reducing sample buffer containing dithiothreitol (DTT) and incubated at room temperature for 2 min. The tubes were centrifuged at 6000 × *g* for 2 min. Finally, the eluted samples were heated for 5 min at 100 °C and subjected to immunoblot analysis. The membrane was incubated with rabbit anti‐RhoA antibody in 10 mL primary antibody dilution buffer with gentle agitation overnight at 4 °C.

### Statistical Analysis

Data are expressed as mean ± standard deviation (s.d.) or standard error of the mean (s.e.m.) from at least three independent results. Statistical difference between the two groups was analyzed by two‐tailed student's *t*‐test while comparisons of multiple groups were evaluated by one‐way analysis of variance (ANOVA) as specified followed by post hoc Tukey's (parametric, equal standard deviation), Dunnett's (parametric, unequal standard deviation), or Dunn's (nonparametric) tests. *p*‐values< 0.05 were considered statistically significant and different numbers of asterisk indicate the levels of significance as follows: *p < 0.05; **p < 0.01; ***p < 0.001; and ****p < 0.0001. Drawing graphs and statistical analyses were performed using GraphPad Prism 8 (GraphPad Software).

## Conflict of Interest

The authors declare no conflict of interest.

## Author Contributions

S.K., A.T., and S.H.K. contributed equally to this work. S.K., A.T., and S.H.K. performed experiments, analyzed the data, and drafted the manuscript. S.J.K., Y.J.K., and M.T. performed experiments. M.Y.K. obtained human study approval and provided human samples. K.Y.O. analyzed the TMA data and provided expertise in cancer pathology. H.S.K. provided biomaterials and experimental support for biomaterials. H.W.K. edited the manuscript, secured funding for the research and supervised the study. J.H. conceptualized the study, designed the experiments, obtained animal study approval, analyzed and interpreted the data, wrote and edited the manuscript, secured funding for the research and supervised the study.

## Supporting information

Supporting Information

Supporting Information

Supporting Information

Supporting Information

Supporting Information

Supporting Information

Supporting Information

Supporting Information

Supplemental Video 1

Supplemental Video 2

Supplemental Video 3

## Data Availability

The data that support the findings of this study are available in the supplementary material of this article.

## References

[1] a) R. Kalluri , Nat. Rev. Cancer 2016, 16, 582; 27550820 10.1038/nrc.2016.73

[2] E. Sahai , I. Astsaturov , E. Cukierman , D. G. DeNardo , M. Egeblad , R. M. Evans , D. Fearon , F. R. Greten , S. R. Hingorani , T. Hunter , Nat. Rev. Cancer 2020, 20, 174. 31980749 10.1038/s41568-019-0238-1 PMC7046529

[3] C. Walker , E. Mojares , A. del Río Hernández , Int. J. Mol. Sci. 2018, 19, 3028. 30287763 10.3390/ijms19103028 PMC6213383

[4] a) B. Rybinski , J. Franco‐Barraza , E. Cukierman , Physiol. Genomics 2014, 46, 223; 24520152 10.1152/physiolgenomics.00158.2013 PMC4035661

[5] a) A. Mazur , E. Holthoff , S. Vadali , T. Kelly , S. R. Post , PLoS One 2016, 11, e0150287; 26934296 10.1371/journal.pone.0150287 PMC4774960

[6] A. Nicolas‐Boluda , J. Vaquero , L. Vimeux , T. Guilbert , S. Barrin , C. Kantari‐Mimoun , M. Ponzo , G. Renault , P. Deptula , K. Pogoda , eLife 2021, 10, e58688. 34106045 10.7554/eLife.58688 PMC8203293

[7] B. L. Bangasser , G. A. Shamsan , C. E. Chan , K. N. Opoku , E. Tüzel , B. W. Schlichtmann , J. A. Kasim , B. J. Fuller , B. R. McCullough , S. S. Rosenfeld , Nat. Commun. 2017, 8, 15313. 28530245 10.1038/ncomms15313 PMC5458120

[8] a) C. Liu , M. Li , Z. X. Dong , D. Jiang , X. Li , S. Lin , D. Chen , X. Zou , X. D. Zhang , G. D. Luker , Acta Biomater. 2021, 131, 326; 34246802 10.1016/j.actbio.2021.07.009 PMC8784164

[9] M. G. Ondeck , A. Kumar , J. K. Placone , C. M. Plunkett , B. F. Matte , K. C. Wong , L. Fattet , J. Yang , A. J. Engler , Proc. Natl. Acad. Sci. USA 2019, 116, 3502. 30755531 10.1073/pnas.1814204116 PMC6397509

[10] a) S. C. Wei , L. Fattet , J. H. Tsai , Y. Guo , V. H. Pai , H. E. Majeski , A. C. Chen , R. L. Sah , S. S. Taylor , A. J. Engler , Nat. Cell Biol. 2015, 17, 678; 25893917 10.1038/ncb3157 PMC4452027

[11] a) R. S. Stowers , A. Shcherbina , J. Israeli , J. J. Gruber , J. Chang , S. Nam , A. Rabiee , M. N. Teruel , M. P. Snyder , A. Kundaje , Nat. Biomed. Eng. 2019, 3, 1009; 31285581 10.1038/s41551-019-0420-5 PMC6899165

[12] Y. Abe , N. Tanaka , BioMed. Res. Int. 2016, 2016, 7969286. 28105432 10.1155/2016/7969286 PMC5220431

[13] A. Otsuka , M. P. Levesque , R. Dummer , K. Kabashima , J. Dermatol. Sci. 2015, 78, 95. 25766766 10.1016/j.jdermsci.2015.02.007

[14] M. E. C. Buim , C. A. S. Gurgel , E. A. G. Ramos , S. V. Lourenço , F. A. Soares , Hum. Pathol. 2011, 42, 1484. 21496866 10.1016/j.humpath.2010.12.015

[15] a) A. Giammona , E. Crivaro , B. Stecca , Int. J. Mol. Sci. 2023, 24, 1321; 36674836 10.3390/ijms24021321 PMC9864846

[16] a) S. J. Matissek , S. F. Elsawa , Cell Commun. Signaling 2020, 18, 54; 10.1186/s12964-020-00540-x PMC7119169 32245491

[17] a) Z. Liu , H. Hayashi , K. Matsumura , Y. Ogata , H. Sato , Y. Shiraishi , N. Uemura , T. Miyata , T. Higashi , S. Nakagawa , Br. J. Cancer 2023, 128, 844; 36536047 10.1038/s41416-022-02106-9 PMC9977781

[18] a) K. Pogoda , M. Cieśluk , P. Deptuła , G. Tokajuk , E. Piktel , G. Król , J. Reszeć , R. Bucki , Transl. Oncol. 2021, 14,101105; 33946032 10.1016/j.tranon.2021.101105 PMC8111093

[19] a) K. Takabatake , T. Shimo , J. Murakami , C. Anqi , H. Kawai , S. Yoshida , M. Wathone Oo , O. Haruka , S. Sukegawa , H. Tsujigiwa , Int. J. Mol. Sci. 2019, 20, 5779; 31744214 10.3390/ijms20225779 PMC6888610

[20] H. Kuroda , N. Kurio , T. Shimo , K. Matsumoto , M. Masui , K. Takabatake , T. Okui , S. Ibaragi , Y. Kunisada , K. Obata , Anticancer Res. 2017, 37, 6731. 29187450 10.21873/anticanres.12132

[21] a) Q. Li , H. Dong , G. Yang , Y. Song , Y. Mou , Y. Ni , Front. Oncol. 2020, 10, 212; 32158692 10.3389/fonc.2020.00212 PMC7052016

[22] a) C. H. Chiang , C. C. Wu , L. Y. Lee , Y. C. Li , H. P. Liu , C. W. Hsu , Y. C. Lu , J. T. Chang , A. J. Cheng , J. Proteome Res. 2016, 15, 2981; 27432155 10.1021/acs.jproteome.6b00138

[23] J. K. Chen , J. Taipale , K. E. Young , T. Maiti , P. A. Beachy , Proc. Natl. Acad. Sci. USA 2002, 99, 14071. 12391318 10.1073/pnas.182542899 PMC137838

[24] Y. Bai , Y. Bai , J. Dong , Q. Li , Y. Jin , B. Chen , M. Zhou , Medicine 2016, 95, e2996. 26962810 10.1097/MD.0000000000002996 PMC4998891

[25] A. Elosegui‐Artola , I. Andreu , A. E. Beedle , A. Lezamiz , M. Uroz , A. J. Kosmalska , R. Oria , J. Z. Kechagia , P. Rico‐Lastres , A. L. Le Roux , Cell 2017, 171, 1397. 29107331 10.1016/j.cell.2017.10.008

[26] a) H. Cao , X. Chen , J. Hou , C. Wang , Z. Xiang , Y. Shen , X. Han , Lab. Invest. 2020, 100, 363; 31541181 10.1038/s41374-019-0316-8

[27] a) N. P. Talele , J. Fradette , J. E. Davies , A. Kapus , B. Hinz , Stem Cell Rep. 2015, 4, 1016; 10.1016/j.stemcr.2015.05.004 PMC4471834 26028530

[28] a) S. Dupont , L. Morsut , M. Aragona , E. Enzo , S. Giulitti , M. Cordenonsi , F. Zanconato , J. L. Digabel , M. Forcato , S. Bicciato , Nature 2011, 474, 179; 21654799 10.1038/nature10137

[29] A. N. Sigafoos , B. D. Paradise , M. E. Fernandez‐Zapico , Cancers 2021, 13, 3410. 34298625 10.3390/cancers13143410 PMC8304605

[30] F. Yin , Q. Chen , Y. Shi , H. Xu , J. Huang , M. Qing , L. Zhong , J. Li , L. Xie , X. Zeng , Oral Dis. 2022, 28, 621. 33529425 10.1111/odi.13791

[31] a) S. Y. Wong , A. D. Seol , P. L. So , A. N. Ermilov , C. K. Bichakjian , E. H. Epstein Jr , A. A. Dlugosz , J. F. Reiter , Nat. Med. 2009, 15, 1055; 19701205 10.1038/nm.2011 PMC2895420

[32] a) J. Ran , Y. Yang , D. Li , M. Liu , J. Zhou , Sci. Rep. 2015, 5, 12917; 26246421 10.1038/srep12917 PMC4526867

[33] H. F. Yee Jr , A. C. Melton , B. N. Tran , Biochem. Biophys. Res. Commun. 2001, 280, 1340. 11162676 10.1006/bbrc.2001.4291

[34] R. Kalluri , R. A. Weinberg , J. Clin. Invest. 2009, 119, 1420. 19487818 10.1172/JCI39104 PMC2689101

[35] a) D. Huang , Y. Wang , L. Xu , L. Chen , M. Cheng , W. Shi , H. Xiong , D. Zalli , S. Luo , J. Exp. Clin. Cancer Res. 2018, 37, 247; 30305138 10.1186/s13046-018-0917-x PMC6180656

[36] A. J. Ridley , Curr. Opin. Cell Biol. 2015, 36, 103. 26363959 10.1016/j.ceb.2015.08.005 PMC4728192

[37] M. E. Fernandez‐Sanchez , S. Barbier , J. Whitehead , G. Béalle , A. Michel , H. Latorre‐Ossa , C. Rey , L. Fouassier , A. Claperon , L. Brullé , Nature 2015, 523, 92. 25970250 10.1038/nature14329

[38] J.‐b. Lin , Z. Feng , M.‐l. Qiu , R.‐g. Luo , X. Li , B. Liu , Future Oncol. 2020, 16, 1903. 32449621 10.2217/fon-2019-0603

[39] Y. Wang , Y. Jing , L. Ding , X. Zhang , Y. Song , S. Chen , X. Zhao , X. Huang , Y. Pu , Z. Wang , J. Exp. Clin. Cancer Res. 2019, 38, 274. 31234944 10.1186/s13046-019-1277-x PMC6591968

[40] a) V. Seewaldt , Nat. Med. 2014, 20, 332; 24710372 10.1038/nm.3523

[41] a) J. Schrader , T. T. Gordon‐Walker , R. L. Aucott , M. van Deemter , A. Quaas , S. Walsh , D. Benten , S. J. Forbes , R. G. Wells , J. P. Iredale , Hepatology 2011, 53, 1192; 21442631 10.1002/hep.24108 PMC3076070

[42] T. Panciera , A. Citron , D. Di Biagio , G. Battilana , A. Gandin , S. Giulitti , M. Forcato , S. Bicciato , V. Panzetta , S. Fusco , Nat. Mater. 2020, 19, 797. 32066931 10.1038/s41563-020-0615-x PMC7316573

[43] a) A. I. Nagasato , H. Yamashita , M. Matsuo , K. Ueda , N. Kioka , Biosci. Biotechnol. Biochem. 2017, 81, 1136; 28485208 10.1080/09168451.2017.1289074

[44] a) M. Mavrakis , M. A. Juanes , Curr. Opin. Cell Biol. 2023, 80, 102152; 36796142 10.1016/j.ceb.2023.102152

[45] B. Cheng , M. Li , W. Wan , H. Guo , G. M. Genin , M. Lin , F. Xu , Biophys. J. 2023, 122, 43. 36451545 10.1016/j.bpj.2022.11.2943 PMC9822792

[46] a) D. Shakiba , F. Alisafaei , A. Savadipour , R. A. Rowe , Z. Liu , K. M. Pryse , V. B. Shenoy , E. L. Elson , G. M. Genin , ACS Nano 2020, 14, 7868; 32286054 10.1021/acsnano.9b09941

[47] a) K. L. Hui , A. Upadhyaya , Proc. Natl. Acad. Sci. USA 2017, 114, E4175; 28490501 10.1073/pnas.1614291114 PMC5448208

[48] Y. Li , O. Kučera , D. Cuvelier , D. M. Rutkowski , M. Deygas , D. Rai , T. Pavlovič , F. N. Vicente , M. Piel , G. Giannone , Nat. Mater. 2023, 22, 913. 37386067 10.1038/s41563-023-01578-1 PMC10569437

[49] a) H. Liu , D. Gu , J. Xie , Chin. J. Cancer 2011, 30, 13; 21192841 10.5732/cjc.010.10540 PMC3137255

[50] G. Brancaccio , F. Pea , E. Moscarella , G. Argenziano , Front. Oncol. 2020, 10, 582866. 33194718 10.3389/fonc.2020.582866 PMC7662670

[51] C. D. Yao , D. Haensel , S. Gaddam , T. Patel , S. X. Atwood , K. Y. Sarin , R. J. Whitson , S. McKellar , G. Shankar , S. Aasi , Nat. Commun. 2020, 11, 5079. 33033234 10.1038/s41467-020-18762-5 PMC7546632

[52] a) M. Cao , X. Zou , C. Li , Z. Lin , N. Wang , Z. Zou , Y. Ye , J. Seemann , B. Levine , Z. Tang , Nat. Commun. 2023, 14, 1687; 36973243 10.1038/s41467-023-37340-z PMC10042869

[53] X. Zhao , E. Pak , K. J. Ornell , M. F. Pazyra‐Murphy , E. L. MacKenzie , E. J. Chadwick , T. Ponomaryov , J. F. Kelleher , R. A. Segal , Cancer Discovery 2017, 7, 1436. 28923910 10.1158/2159-8290.CD-17-0281

[54] a) J. J. Doyle , E. E. Gerber , H. C. Dietz , FEBS Lett. 2012, 586, 2003; 22641039 10.1016/j.febslet.2012.05.027 PMC3426037

[55] a) M. Chirivì , F. Maiullari , M. Milan , D. Presutti , C. Cordiglieri , M. Crosti , M. L. Sarnicola , A. Soluri , M. Volpi , W. Święszkowski , Int. J. Mol. Sci. 2021, 22, 5862; 34070750 10.3390/ijms22115862 PMC8198248

[56] a) C. Feig , J. O. Jones , M. Kraman , R. J. Wells , A. Deonarine , D. S. Chan , C. M. Connell , E. W. Roberts , Q. Zhao , O. L. Caballero , Proc. Natl. Acad. Sci. USA 2013, 110, 20212; 24277834 10.1073/pnas.1320318110 PMC3864274

[57] a) R. S. O'Connor , X. Hao , K. Shen , K. Bashour , T. Akimova , W. W. Hancock , L. C. Kam , M. C. Milone , J. Immunol. 2012, 189, 1330; 22732590 10.4049/jimmunol.1102757 PMC3401283

[58] J. Hyun , S. J. Kim , S. D. Cho , H. W. Kim , Biomaterials 2023, 297, 122101. 37023528 10.1016/j.biomaterials.2023.122101

[59] I. Caruana , B. Savoldo , V. Hoyos , G. Weber , H. Liu , E. S. Kim , M. M. Ittmann , D. Marchetti , G. Dotti , Nat. Med. 2015, 21, 524. 25849134 10.1038/nm.3833 PMC4425589

[60] Y. Chen , S. Zhu , T. Liu , S. Zhang , J. Lu , W. Fan , L. Lin , T. Xiang , J. Yang , X. Zhao , Cancer Cell 2023, 41, 903. 36963399 10.1016/j.ccell.2023.03.001

[61] K. Kappert , N. Connesson , S. Elahi , S. Boonstra , A. Balm , F. van Der Heijden , Y. Payan , J. Biomech. 2021, 114, 110147. 33276256 10.1016/j.jbiomech.2020.110147

[62] M. Shibata , A. Ishikawa , J. Ishii , E. Anzai , H. Yagishita , T. Izumo , J. Sumino , M. Katsurano , Y. Kim , H. Kanda , Oral Surg. Oral Med. Oral Pathol. Oral Radiol. 2023, 135, 558. 36535887 10.1016/j.oooo.2022.11.001

[63] a) A. Buchholz , A. Vattai , S. Fürst , T. Vilsmaier , C. Kuhn , E. Schmoeckel , D. Mayr , C. Dannecker , S. Mahner , U. Jeschke , Cancers 2021, 13, 1410; 33808776 10.3390/cancers13061410 PMC8003514

[64] F. Grizzi , S. Fiorino , D. Qehajaj , A. Fornelli , C. Russo , D. De Biase , M. Masetti , L. Mastrangelo , M. Zanello , R. Lombardi , J. Transl. Med. 2019, 17, 61. 30819202 10.1186/s12967-019-1817-3 PMC6393991

[65] S. R. Caliari , M. Perepelyuk , E. M. Soulas , G. Y. Lee , R. G. Wells , J. A. Burdick , Integr. Biol. 2016, 8, 720. 10.1039/c6ib00027d PMC4905794 27162057

