# Supplementary material for: Tissue Mechanics and Hedgehog Signaling Crosstalk as a Key Epithelial–Stromal Interplay in Cancer Development
Source: Adv Sci (Weinh). 2024 Jul 8;11(35):2400063. doi: 10.1002/advs.202400063 (PMC11425211; doi:10.1002/advs.202400063)
Supplement: Supplementary file 1 — Supporting Information [file ADVS-11-2400063-s001.docx]

**Tissue Mechanics and Hedgehog Signaling Crosstalk as a Key Epithelial-Stromal Interplay in Cancer Development**

*Shanika Karunasagara^1,2,#^, Ali Taghizadeh^1,2,#^, Sang-Hyun Kim^1,3,#^, So Jung Kim^1,2^, Yong-Jae Kim^1,2^, Mohsen Taghizadeh^1,2^, Moon-Young Kim^4^, Kyu-Young Oh^5^, Jung-Hwan Lee^1,2,6,7,8,10^, Hye Sung Kim^1,2,6,7^, Jeongeun Hyun^1,2,6,7,9,*^, Hae-Won Kim^1,2,6,7,9,10,*^*

^1^Institute of Tissue Regeneration Engineering (ITREN) | ^2^Department of Nanobiomedical Science & BK21 Global Research Center for Regenerative Medicine | ^3^Department of Chemistry, College of Science & Technology | ^4^Department of Oral and Maxillofacial Surgery, College of Dentistry | ^5^Department of Oral Pathology, College of Dentistry | ^6^Mechanobiology Dental Medicine Research Center | ^7^UCL Eastman-Korea Dental Medicine Innovation Centre | ^8^Department of Biomaterials Science, College of Dentistry | ^9^Department of Regenerative Dental Medicine, College of Dentistry | ^10^Cell & Matter Institute, Dankook University, Cheonan 31116, Republic of Korea.

^#^These authors contributed equally to this work.

^*^Corresponding authors:

Jeongeun Hyun (j.hyun@dankook.ac.kr) & Hae-Won Kim (kimhw@dku.edu)

119, Dandae-ro, Dongnam-gu, Cheonan 31116, Republic of Korea

Tel. +82-41-550-3082

**Supporting Information**


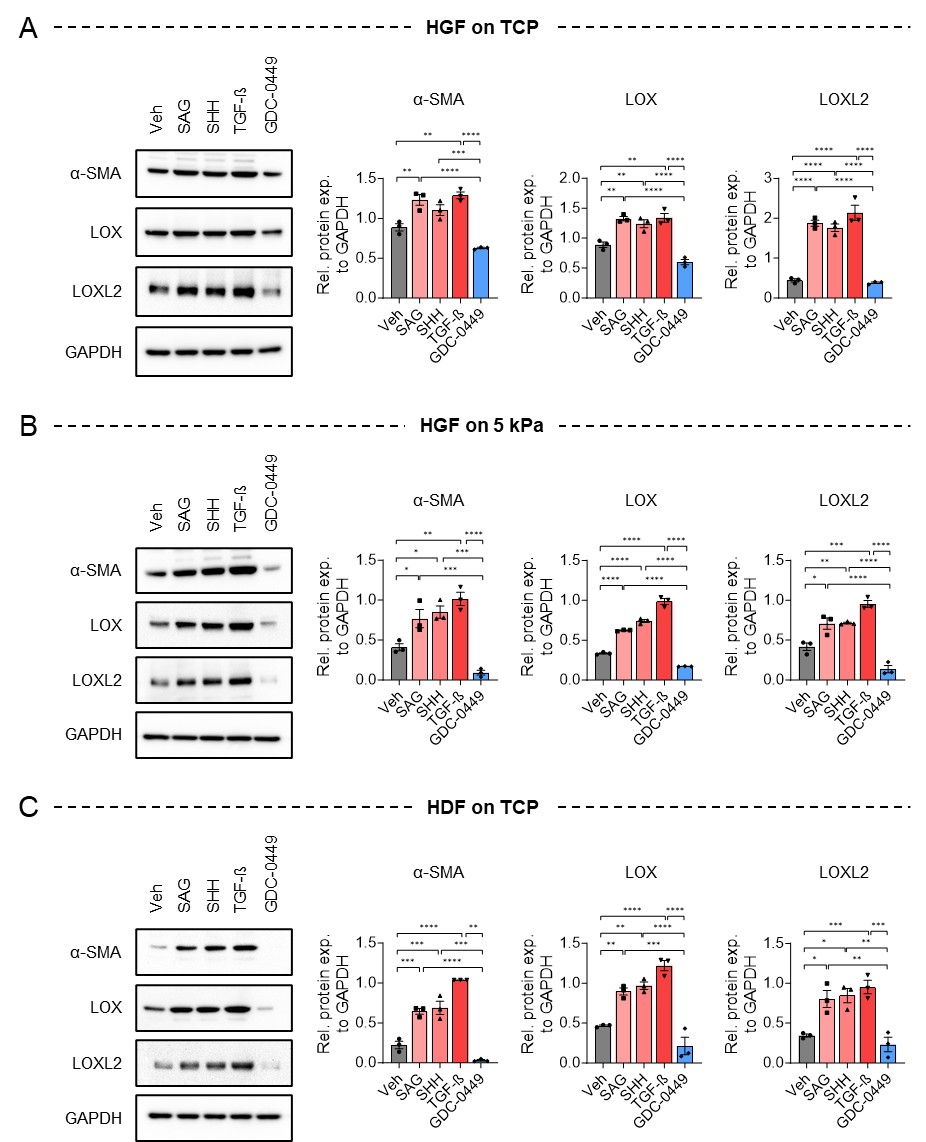


**Figure S1. Sonic Hedgehog (SHH) is released from epithelial cells during chronic tongue/esophageal injury and promotes the activation of myofibroblasts. (A-C)** Immunoblots for α-SMA, LOX, LOXL2, and GAPDH as a loading control in HGF cells cultured either on a tissue culture plate (TCP) (A) or a 5 kPa hydrogel (B) under the treatment with SAG, SHH, TGF-β, or GDC-0449 for 24 h compared to the vehicle (Veh). For a validation, HDF cells were also used (C). The data are presented as mean ± standard error of mean (s.e.m.). Statistical analysis involved one-way ANOVA followed by *post hoc* Tukey’s test. Statistical significance was considered for *p*-values$<$0.05, indicated by asterisks (*, *p*$<$0.05; **, *p*$<$0.01; ***, *p*$<$0.001; and ****, *p*$<$0.0001).

**
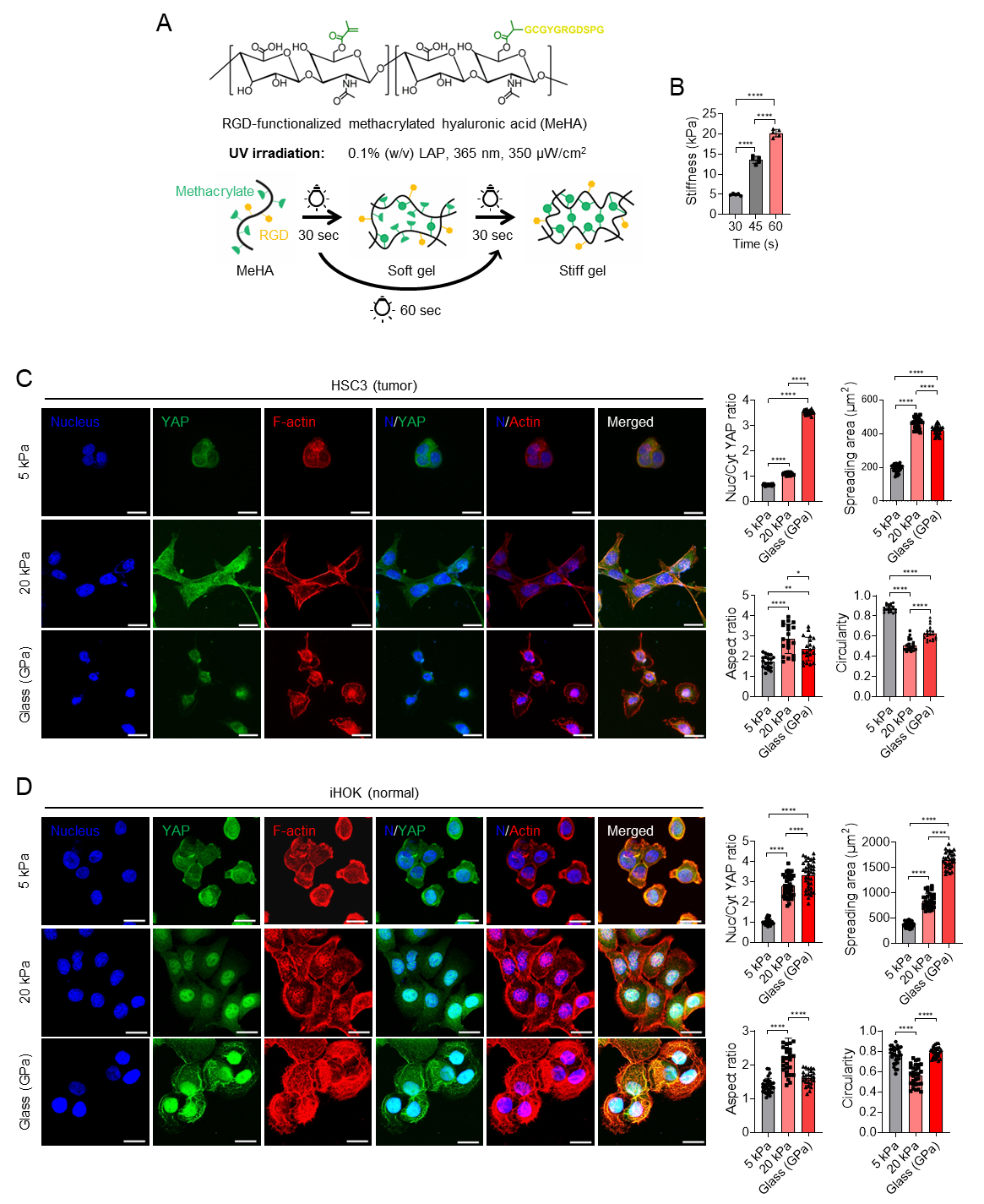
**

**Figure S2. Synthesis of methacrylated hyaluronic acid (MeHA) hydrogels and evaluation of mechanoresponsivenss in oral epithelial cells.** **(A)** A schematic illustration of the chemical structure of RGD (Arg-Gly-Asp)-functionalized methacrylated hyaluronic acid (MeHA) and the preparation procedure for synthesizing soft (5 kPa) and stiff (20 kPa) MeHA hydrogels with static mechanical properties through UV photocrosslinking. **(B)** The stiffness, expressed in kilopascals (kPa), for soft (5 kPa), intermediate, and stiff (20 kPa) MeHA hydrogels with different UV exposure times. **(C, D)** Representative confocal images depicting immunofluorescent (IF) staining for nuclei (blue), YAP (green), and F-actin (red) in HSC3 **(C)** or iHOK **(D)** cells cultured on soft (5 kPa), stiff (20 kPa) hydrogels, or a glass substrate ($\approx$GPa). The nuclear to cytoplasmic ratios (Nuc/Cyt) of YAP expression were quantified. Scale bar = 20 µm. Additionally, cellular morphological parameters, including spreading area, aspect ratio, and circularity, were analyzed. The data are presented as mean $\pm$ standard deviation (s.d.). Statistical analysis involved one-way ANOVA followed by *post hoc* Tukey’s test. Statistical significance was considered for *p*-values$<$0.05, indicated by asterisks (*, *p*$<$0.05; **, *p*$<$0.01; ***, *p*$<$0.001; and ****, *p*$<$0.0001).


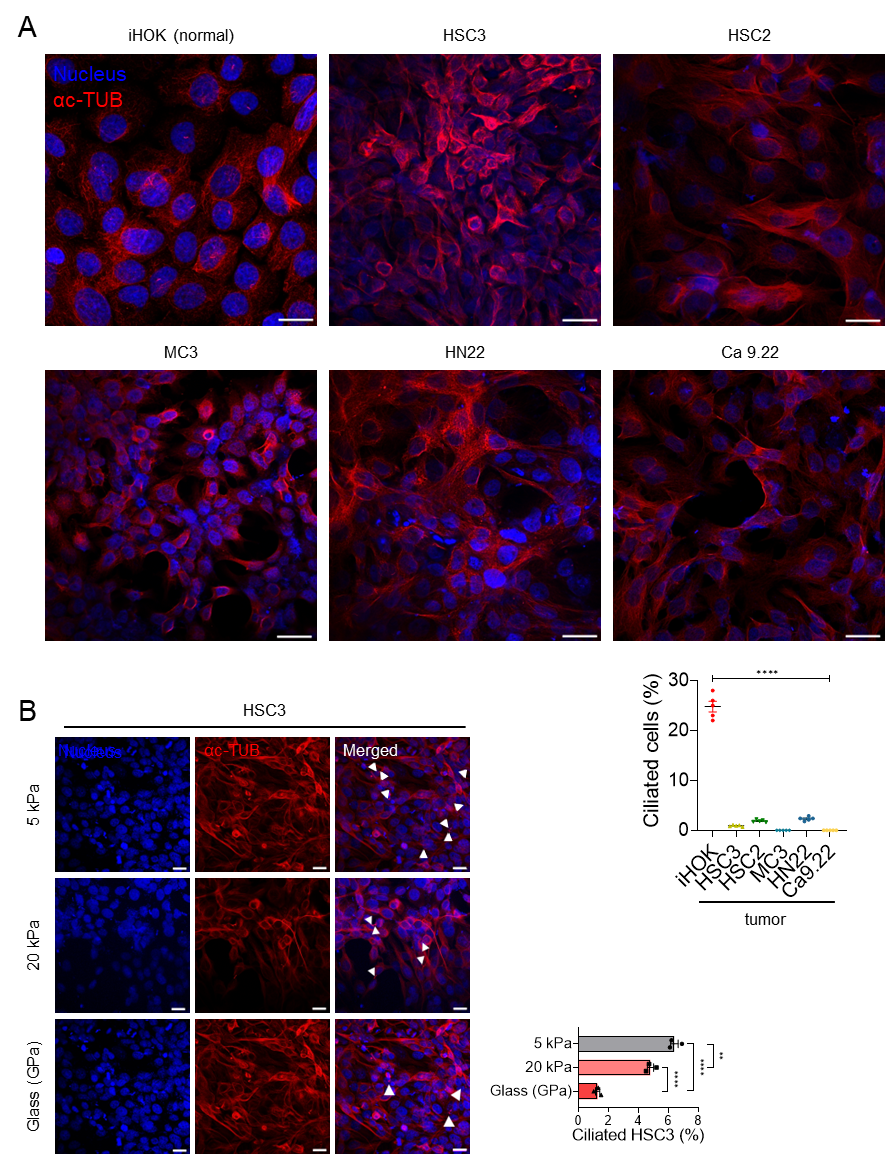


**Figure S3. Oral squamous cell carcinoma (OSCC) cells lose primary cilia (PCs) on stiff matrices. (A)** Representative confocal images illustrating IF staining for nuclei (blue) and acetylated α-Tubulin (ac-TUB, red) in iHOK and five different OSCC cell lines (HSC3, HSC2, MC3, HN22, and Ca 9.22) cultured on confocal dishes. The cells with a primary cilium were quantified. Scale bar = 20 µm. Statistical analysis involved unpaired *t*-tests with Welch’s correction to compare between iHOK cells and each OSCC cell line. **(B)** Representative confocal images and quantification demonstrating the presence of ciliated cells detected using IF for acetylated α-Tubulin (red) in HSC3 cells cultured on soft (5 kPa) or stiff (20 kPa) hydrogels, or a glass substrate. Scale bar = 20 µm. Statistical analysis involved one-way ANOVA followed by *post hoc* Tukey’s test. The mean $\pm$ s.e.m. results are presented and statistically significance was considered for *p*-values$<$0.05, indicated by asterisks (*, *p*$<$0.05; **, *p*$<$0.01; ***, *p*$<$0.001; and ****, *p*$<$0.0001).


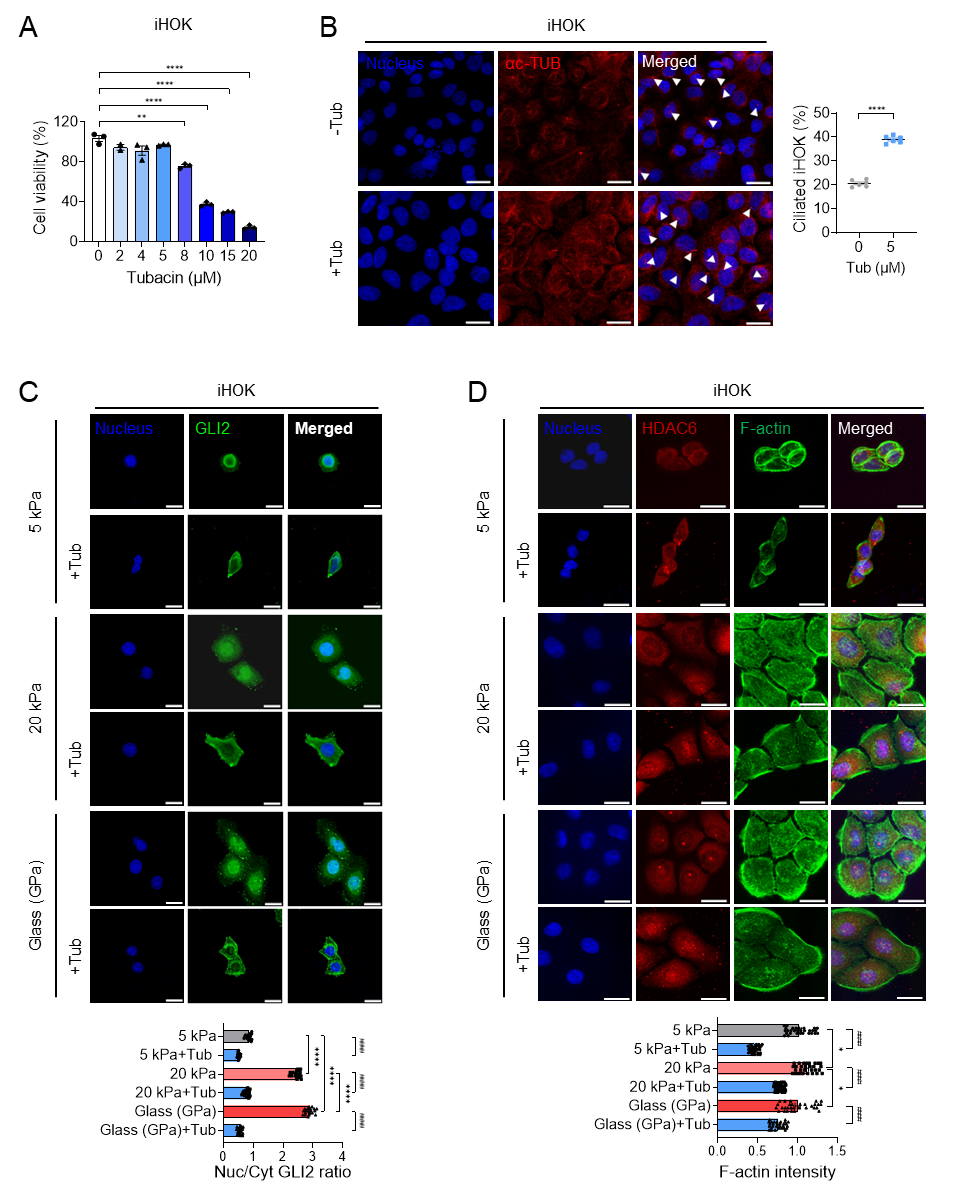


**Figure S4. Treatment with tubacin inhibits GLI2 nuclear translocation through a decrease in filamentous actin (F-actin) while increasing ciliated cells.** **(A)** Cell viability analysis at 24 h after tubacin (an inhibitor of the α-tubulin deacetylating activity of HDAC6) treatment in iHOK cells. Unpaired *t*-tests were used to compare cell viability before tubacin treatment with cell viability after tubacin treatment at each concentration. **(B)** Representative confocal images and quantification demonstrating the presence of ciliated cells detected using IF for acetylated α-Tubulin (ac-TUB, red) in iHOK cells with (+Tub) and without (-Tub) tubacin treatment. An unpaired *t*-test was performed between the two groups. **(C)** Representative confocal images and quantification demonstrating the nuclear to cytoplasmic ratio (Nuc/Cyt) of GLI2 (green) in iHOK cells cultured on different substrate stiffnesses with or without tubacin treatment. One-way ANOVA followed by *post hoc* Tukey’s test was conducted to compare the different stiffness groups. Additionally, unpaired *t*-tests with Welch’s correction were used to compare the tubacin-treated group with corresponding stiffness-matched tubacin-untreated group. **(D)** Representative confocal images of IF for HDAC6 (red) and F-actin (green) in iHOKs cells cultured on different substrate stiffnesses with or without tubacin treatment. F-actin intensities in iHOK cells were quantified. DAPI (blue) was employed as the nuclear staining. Scale bar = 20 µm. The mean $\pm$ s.e.m. results are presented, and statistically significance was considered for *p*-values$<$0.05, indicated by asterisks (**, *p*$<$0.01; ****, *p*$<$0.0001; and ^####^, *p*$<$0.0001).

**
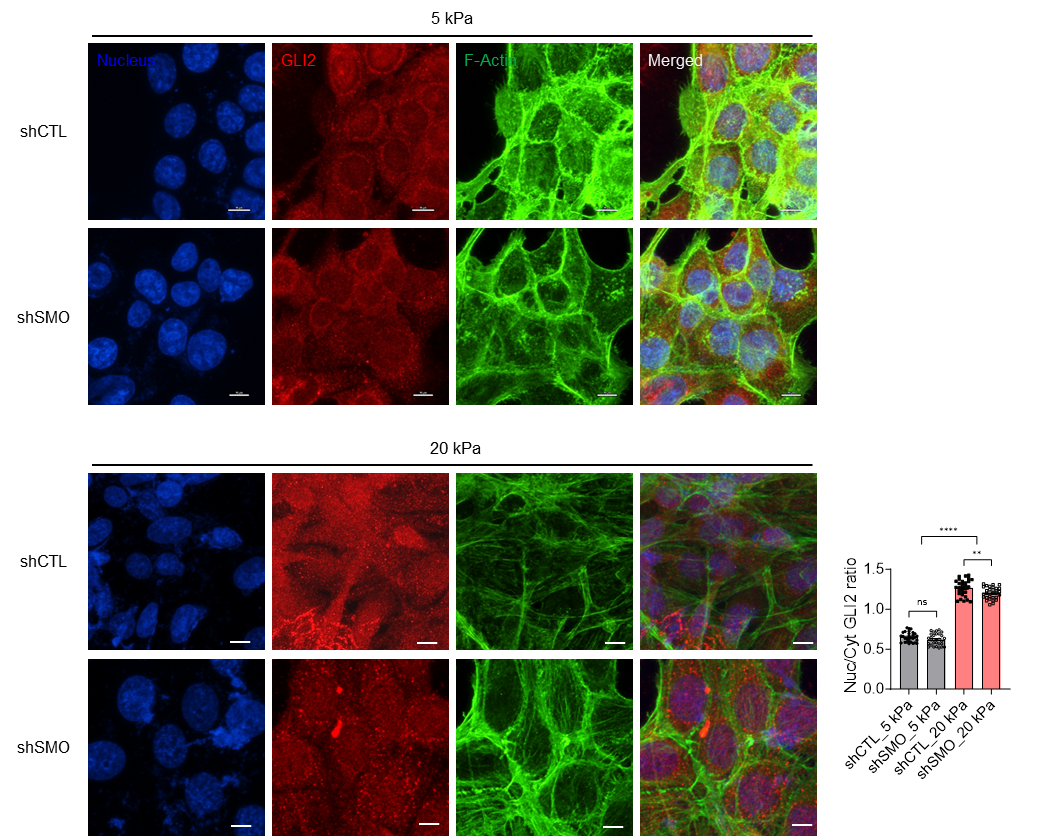
**

**Figure S5. Nuclear localization of GLI2 is maintained under high stiffness condition without canonical Hh signaling.** Representative confocal images illustrating nuclei (blue), GLI2 (red), F-actin (green) in HSC3 cells cultured on soft (5 kPa) or stiff (20 kPa) hydrogels. HSC3 cells were transduced with lentiviral shRNA for Smo (shSMO) to deplete the *Smo* gene or a negative control shRNA (shCTL). The nuclear to cytoplasmic ratio (Nuc/Cyt) of GLI2 expression was quantified. Scale bar = 10 µm. The mean $\pm$ s.e.m. results are presented. Statistical analysis involved two-way ANOVA followed by *post hoc* Fisher’s LSD for multiple group comparisons (**, *p*$<$0.01; and ****, *p*$<$0.0001).


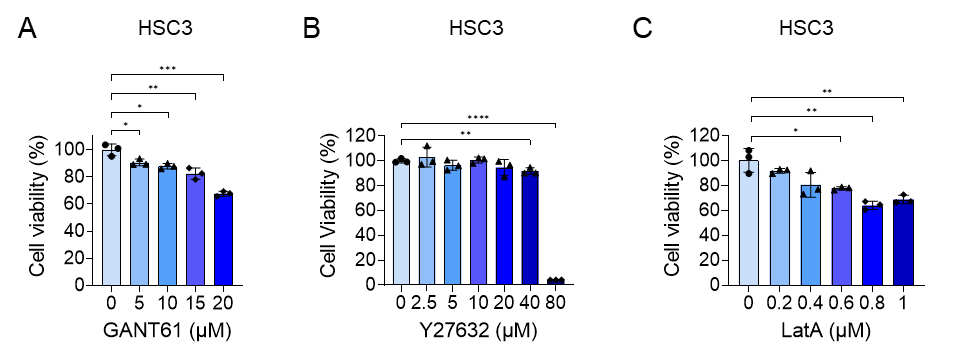


**Figure S6. Evaluation of cell viability following treatment with inhibitor. (A-C)** Cell viability analyses at 24 h after treatment with GANT61 (an inhibitor of GLI2, **A**), Y27632 (a selective Rho kinase inhibitor, **B**), or LatA (an inhibitor of actin polymerization, **C**) in HSC3 cells. The mean $\pm$ s.d. results are presented. Unpaired *t*-tests were performed to compare cell viability before and after inhibitor treatment at each concentration. Statistically significance was considered for *p*-values$<$0.05, indicated by asterisks (*, *p*$<$0.05; **, *p*$<$0.01; ***, *p*$<$0.001; and ****, *p*$<$0.0001).


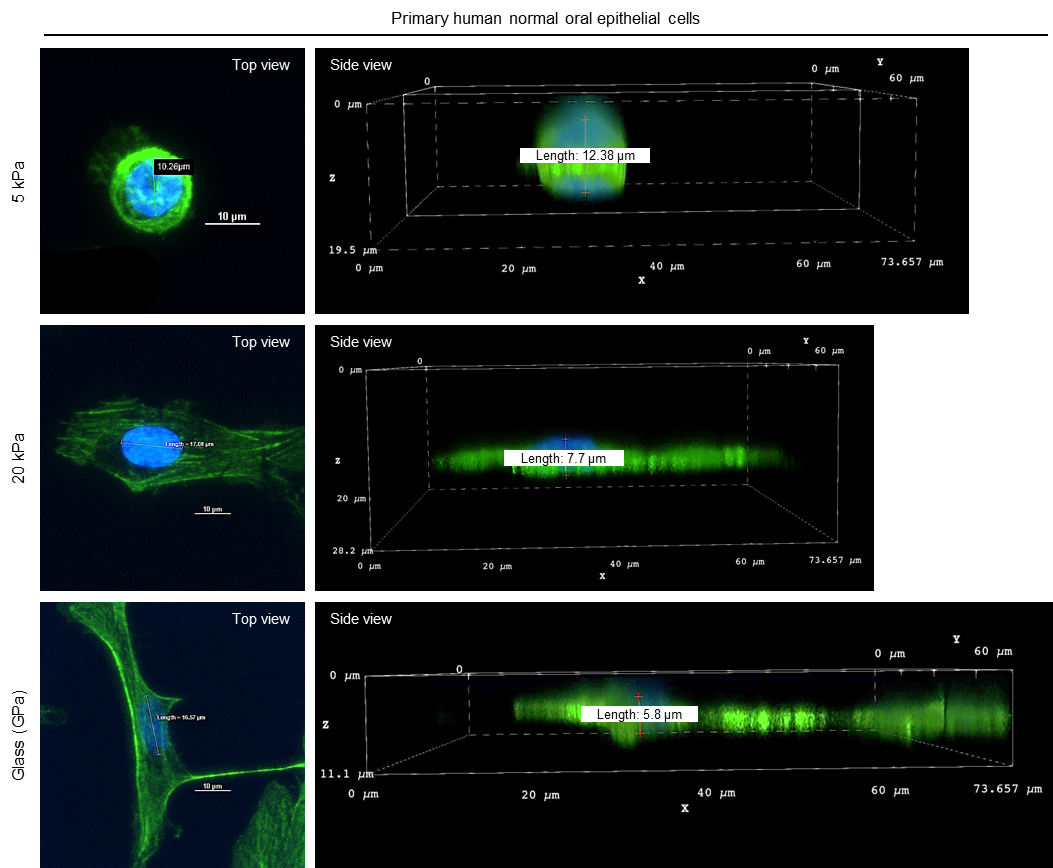


**Figure S7. Increased matrix stiffness induces nuclear flattening of primary oral epithelial cells.** Representative images of primary oral epithelial cells isolated from human normal gingival mucosal tissues, cultured on either soft (5 kPa) or stiff (20 kPa) hydrogels or glass bottom dishes (~GPa), and stained for F-actin (green) and nuclei (blue) were shown. The calculated nuclear flattening indexes were shown in Figure 2F.


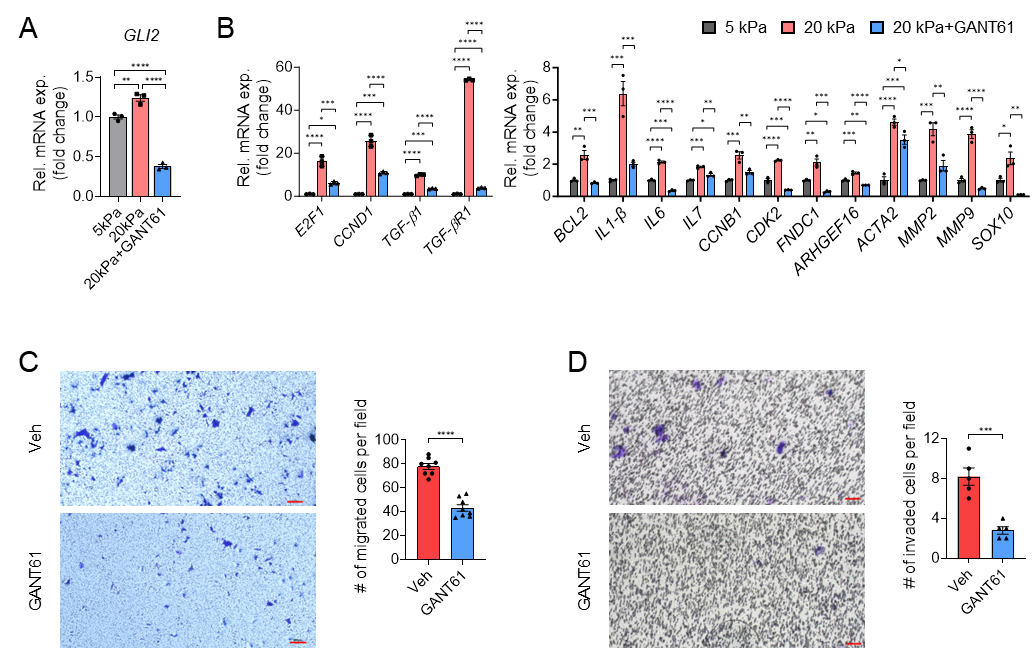


**Figure S8. Mechanically activated GLI2 promotes cell proliferation, migration, and invasion of OSCC cells. (A, B)** qRT-PCR analysis of *GLI2* **(A)**, proliferation marker genes (*CCND1*, *CCNB1*, *CDK2*), mesenchymal marker genes (*ACTA2*, *MMP2*, *MMP9*, *SOX10*), and GLI2-associated or GLI2 target genes (*E2F1*, *TGF-β1*, *TGF-β1R*, *BCL2*, *IL1-β*, *IL6*, *IL7*, *FNDC1*, *ARHGEF16*) **(B)** in HSC3 cells cultured on soft (5 kPa) or stiff (20 kPa) hydrogels with or without treatment with GANT61 (15 µM). One-way ANOVA followed by *post hoc* Tukey’s test was conducted to compare the three conditions. **(C, D)** Representative bright field images of migrated **(C)** and invaded **(D)** HSC3 cells treated with vehicle (Veh) or GANT61. Scale bars = 200 µm. The number of migrated or invaded cells per field was quantified, and unpaired *t*-tests were used to compare between Veh- and GANT61-treated HSC3 cells. The mean $\pm$ s.e.m. results are presented, and statistically significance was considered for *p*-values$<$0.05, indicated by asterisks (*, *p*$<$0.05; **, *p*$<$0.01; ***, *p*$<$0.001; and ****, *p*$<$0.0001).


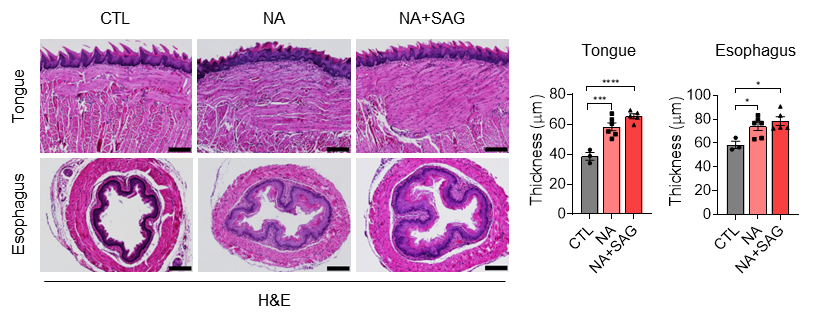


**Figure S9. Epithelial thickness is increased in the tongue and esophageal tissues of a mouse model of chronic tongue/esophageal injury.** Hematoxylin and eosin (H&E) staining in the tongue and esophageal tissue sections of CTL, NA, and NA+SAG mice. Representative images are displayed with scale bars of 200 μm. The epithelial thicknesses of the tongue and esophagus were measured, and the data are presented as mean ± s.e.m. from all individuals in each group. Statistical analysis involved one-way ANOVA followed by *post hoc* Tukey’s test. Statistically significance was considered for *p*-values$<$0.05, denoted by asterisks (*, *p*$<$0.05; **, *p*$<$0.01; ***, *p*$<$0.001; and ****, *p*$<$0.0001).

**
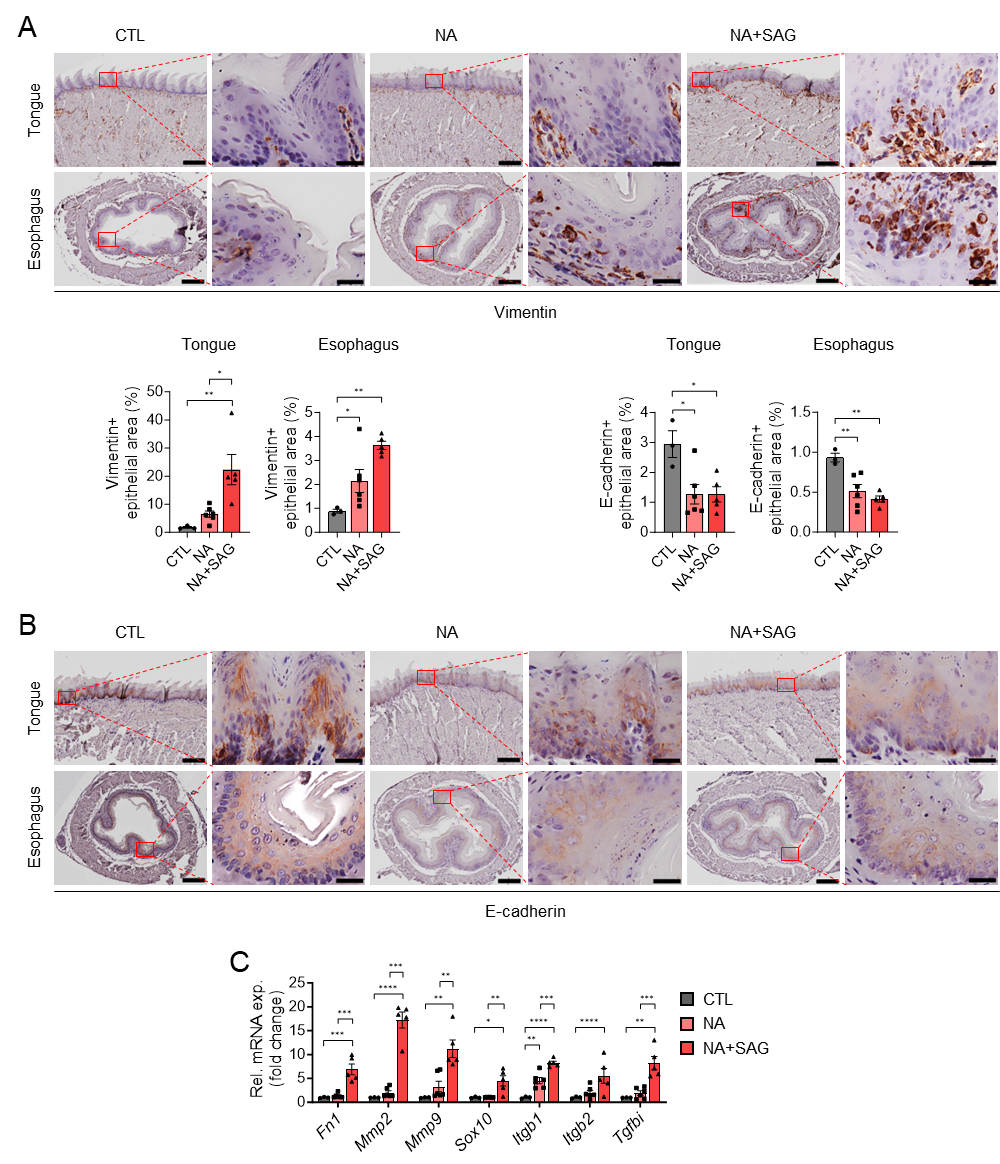
**

**Figure S10. Epithelial cells undergo epithelial-to-mesenchymal transition during tongue/esophageal epithelial dysplasia.** **(A, B)** IHC for Vimentin+ **(A)** or E-cadherin+ **(B)** epithelial cells and the area in the tongue and esophagus of the mice. Scale bar = 200 µm, 20 µm (inset). **(C)** qRT-PCR analysis for the expression of mesenchymal markers (*Fn1, Mmp2, Mmp9, Sox10, Itgb1, Itgb2, Tgfbi*). The mean $\pm$ s.e.m. results are displayed. Statistical analysis involved one-way ANOVA followed by *post hoc* Tukey’s or Dunnett’s test for multiple group comparisons (*, *p*$<$0.05; **, *p*$<$0.01; ***, *p*$<$0.001; and ****, *p*$<$0.0001). Representative images are shown.

**
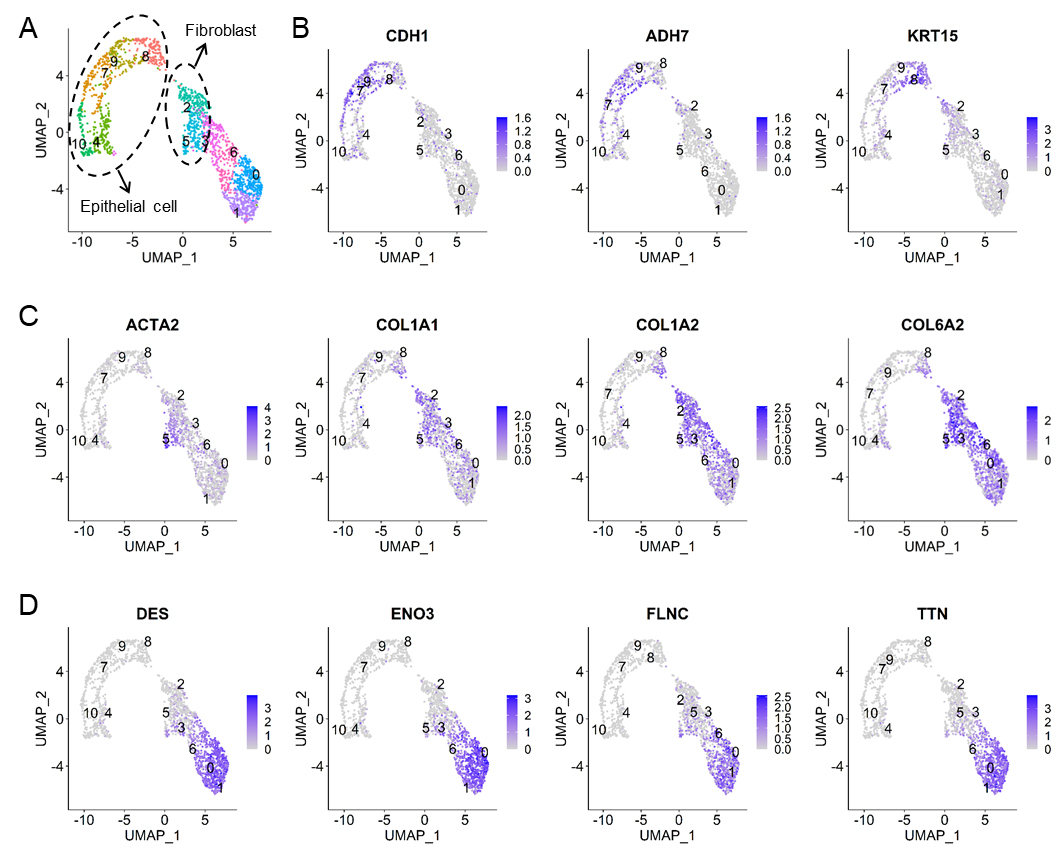
**

**Figure S11. Validation of cluster assignment for spatial transcriptomics analysis by plotting the expression of marker genes in UMAP. (A)** The UMAP embedding of spatial transcriptomics, with different colors representing the assigned clusters. Clusters 4, 7-10 correspond to epithelial cell clusters, while clusters 2, 3, and 5 represent fibroblast clusters. These clusters are highlighted using dotted circles for clarity. **(B)** Feature plots are used to visualize the expression of key epithelial cell marker genes, such as *CDH1*, *ADH7*, and *KRT15*. The expression levels are depicted with color mapping, where grey indicates negative expression and purple indicates high expression. The expression of **(C)** fibroblast marker genes, including *ACTA2*, *COL1A1*, *COL1A2*, and *COL6A2*, and **(D)** muscle cell marker genes, including *DES*, *ENO3*, *FLNC*, and *TTN*, is shown using feature plots.


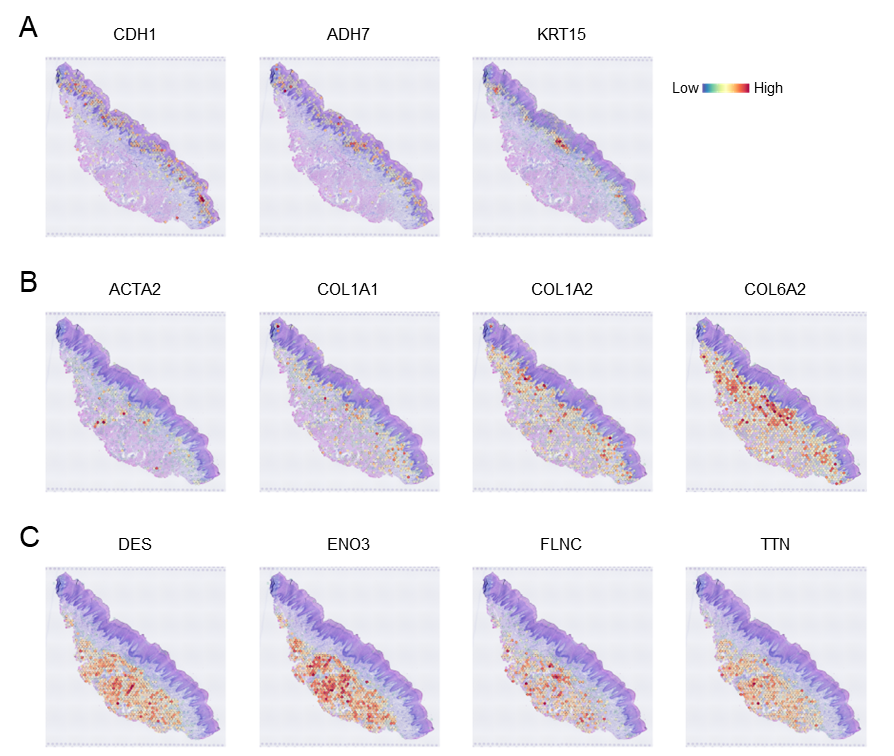


**Figure S12. Validation of cluster assignment for spatial transcriptomics analysis by plotting the expression of marker genes in tissue coordinates. (A-C)** The expression of marker genes specific to epithelial cells **(A)**, fibroblasts **(B)**, and muscle cells **(C)** is projected onto the tissue, showing a color gradient from low (blue) to high (red) to represent the expression levels of these genes.


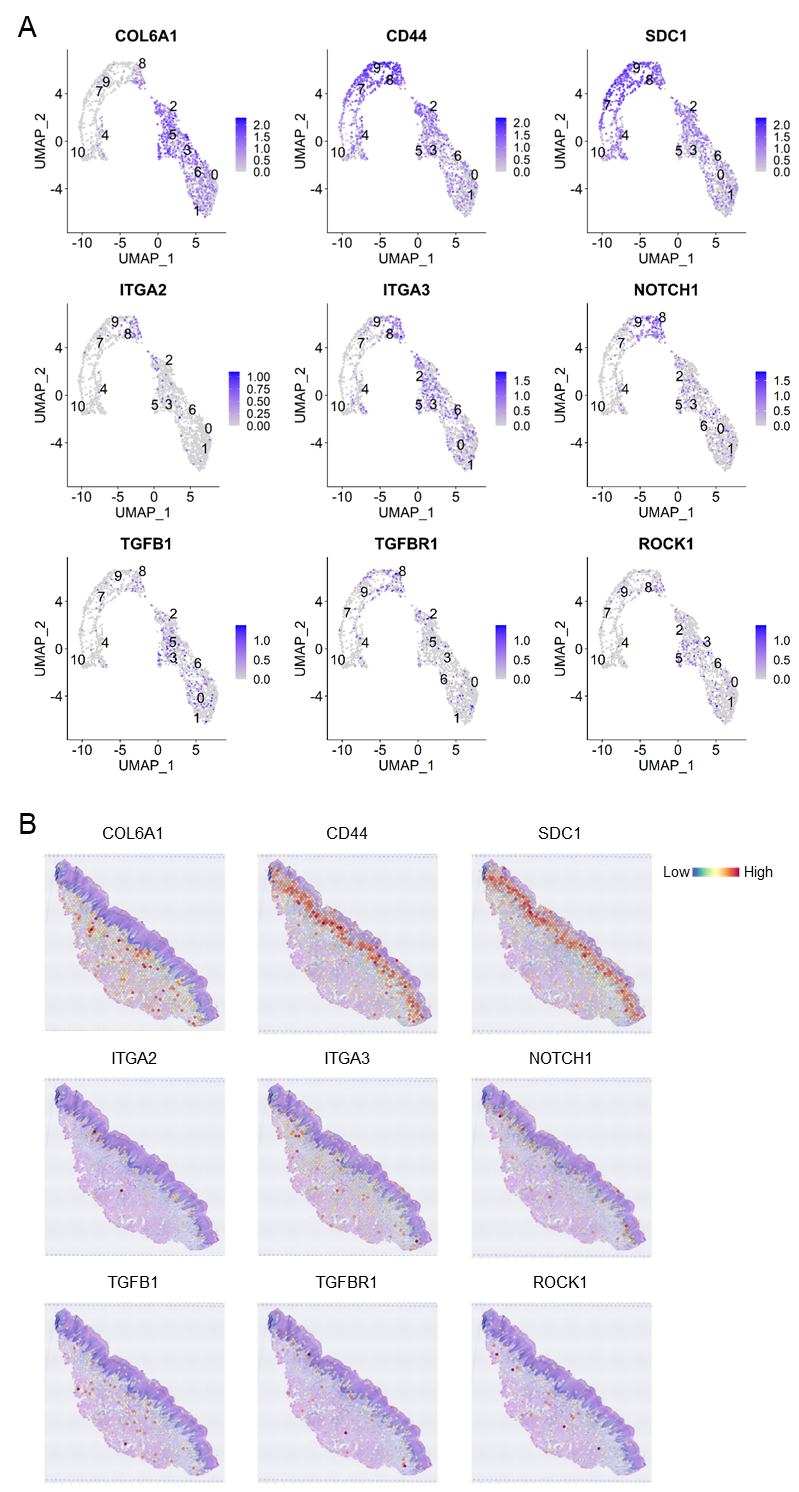


**Figure S13. The expression levels and spatial distributions of genes associated with cell-substrate adhesion and cancer-related genes in the spatial transcriptomics dataset. (A)** Feature plots show the expression of specific genes of interest, including *COL6A1*, *CD44*, *SDC1*, *ITGA2*, *ITGA3*, *NOTCH1*, *TGFB1*, *TGFBR1*, and *ROCK1*, in this study. **(B)** Those selected genes from the spatial transcriptomics analysis dataset are projected onto the tissue space to show their spatial distribution and expression levels.

**
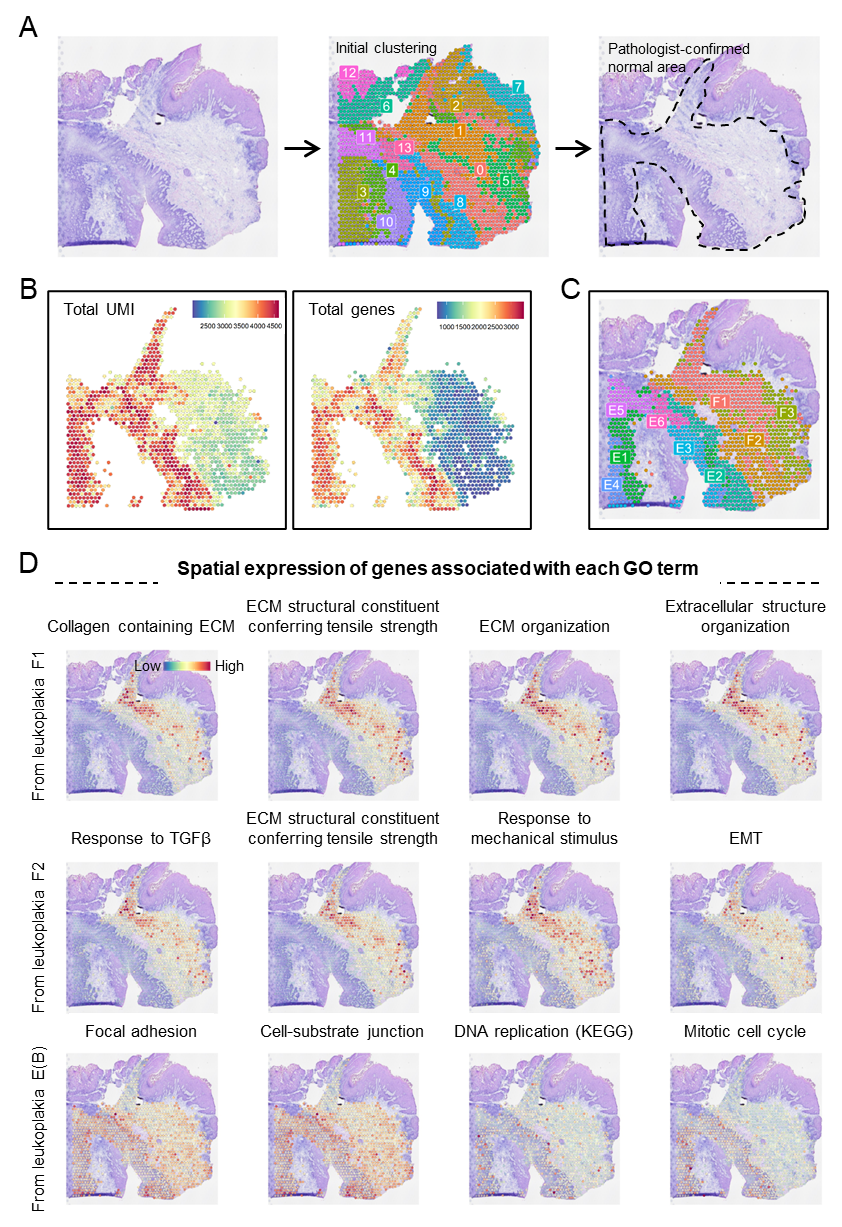
**

**Figure S14. Few mechanobiological interactions were observed between epithelial cells and fibroblasts in normal human oral mucosal tissue. (A)** After initial clustering, only pathologist-confirmed normal-like clusters were analyzed further, as the normal tissue used in this study exhibited some abnormal histological changes and inflammation, despite not being diagnosed as oral disease or pathological condition and being excised during the third molar extraction. The pathologist-confirmed normal tissue area was indicated. **(B)** The Visium array spots on the tissue section are color-coded based on the number of normalized unique molecular identifiers (UMIs, left) or total genes (right) in the dataset. **(C)** The Visium array spots are colored based on the clustering assignments generated from the dataset. Six clusters were identified as epithelial cells, and three clusters were identified as fibroblasts. **(D)** The average standardized expression of leukoplakia-derived gene sets associated with annotated gene ontology (GO) terms was depicted, with expression levels ranging from low (blue) to high (red).

**
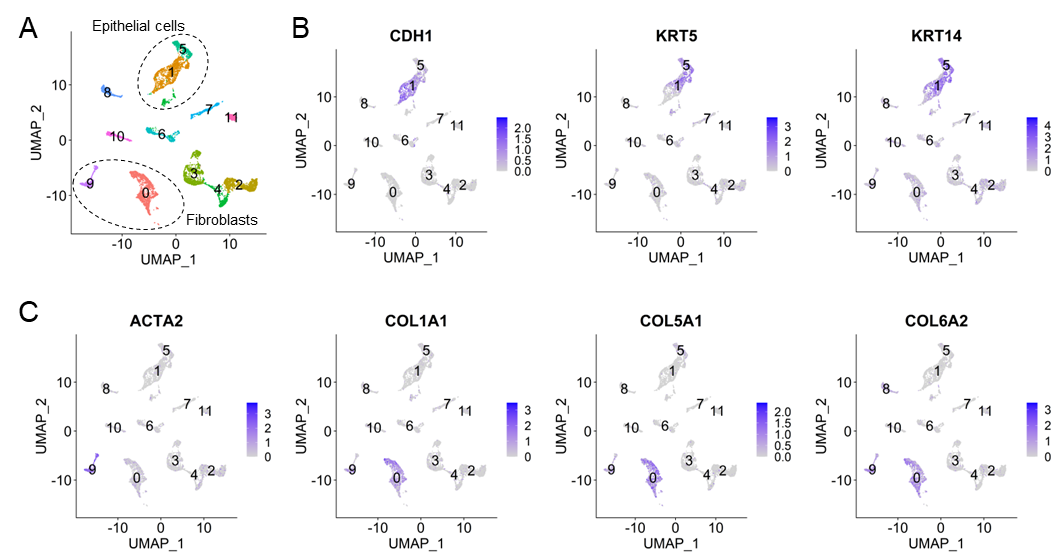
**

**Figure S15. Validation of cluster assignment for snRNA-seq analysis by plotting the expression of marker genes in UMAP. (A)** The UMAP plot displays the snRNA-seq cluster assignments. **(B-C)** The expression of marker genes specific to epithelial cells, such as *CDH1*, *KRT5*, and *KRT14* **(B)**, and fibroblasts, including *ACTA2*, *COL1A1*, COL5A1, and *COL6A2* **(C)**, is shown in the snRNA-seq dataset.


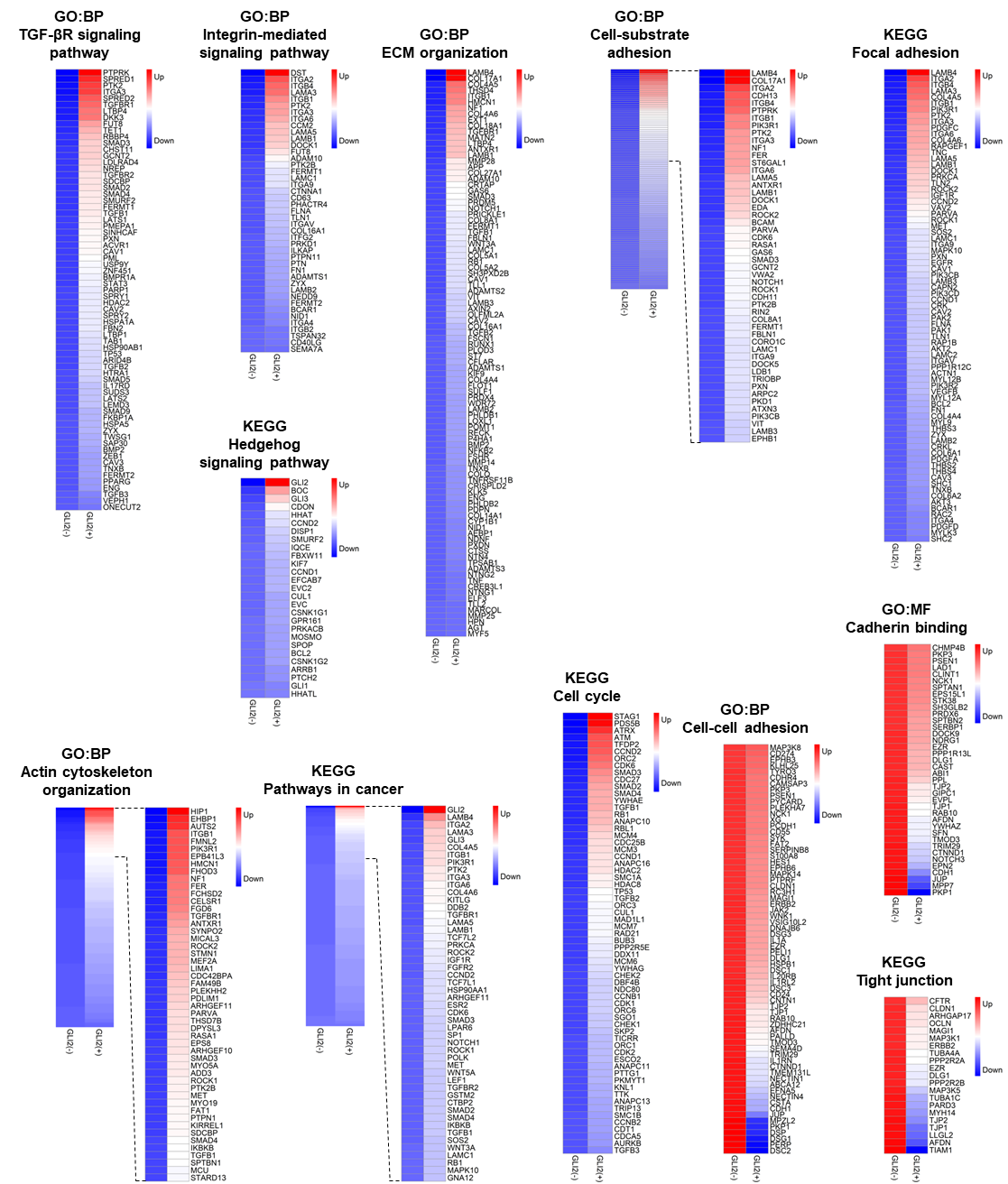


**Figure S16. Functional annotation of the gene profile of GLI2-positive epithelial cells compared to GLI2-negative epithelial cells.** Heatmaps display the expression of genes associated with GO terms and KEGG pathways that are enriched in GLI2-positive epithelial cells when compared to GLI2-negative epithelial cells. The color gradient in the heatmap represents the expression levels, ranging from downregulated (blue) to upregulated (red) expression specifically in GLI2-expressing cells.


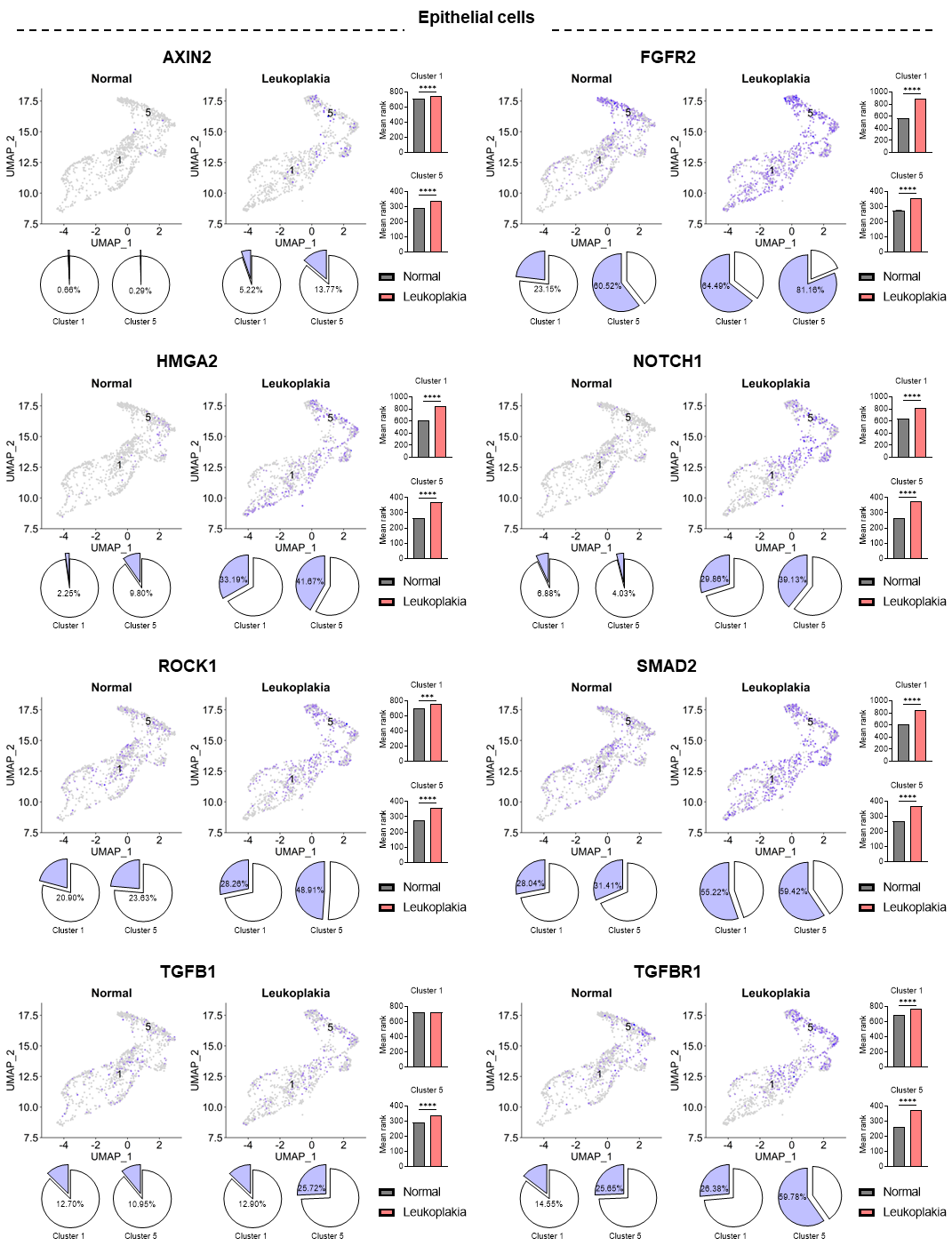


**Figure S17. Upregulation of cancer-related genes in epithelial cells of leukoplakia tissue compared to cells of normal tissue.** The expression of cancer-related genes, including *AXIN2*, *FGFR2*, *HMGA2*, *NOTCH1*, *ROCK1*, *SMAD2*, *TGFB1*, and *TGFBR1*, is visualized for epithelial cell clusters (cluster 1 and 5) in the snRNA-seq dataset. The pie charts provide information on the percentage of positive nuclei for each gene within the respective epithelial cell clusters. The bar plots indicate the mean rank$\pm$s.e.m. for gene expression. Statistical significance was assessed using the Mann-Whitney U test, with a *p*-value threshold of less than 0.05.


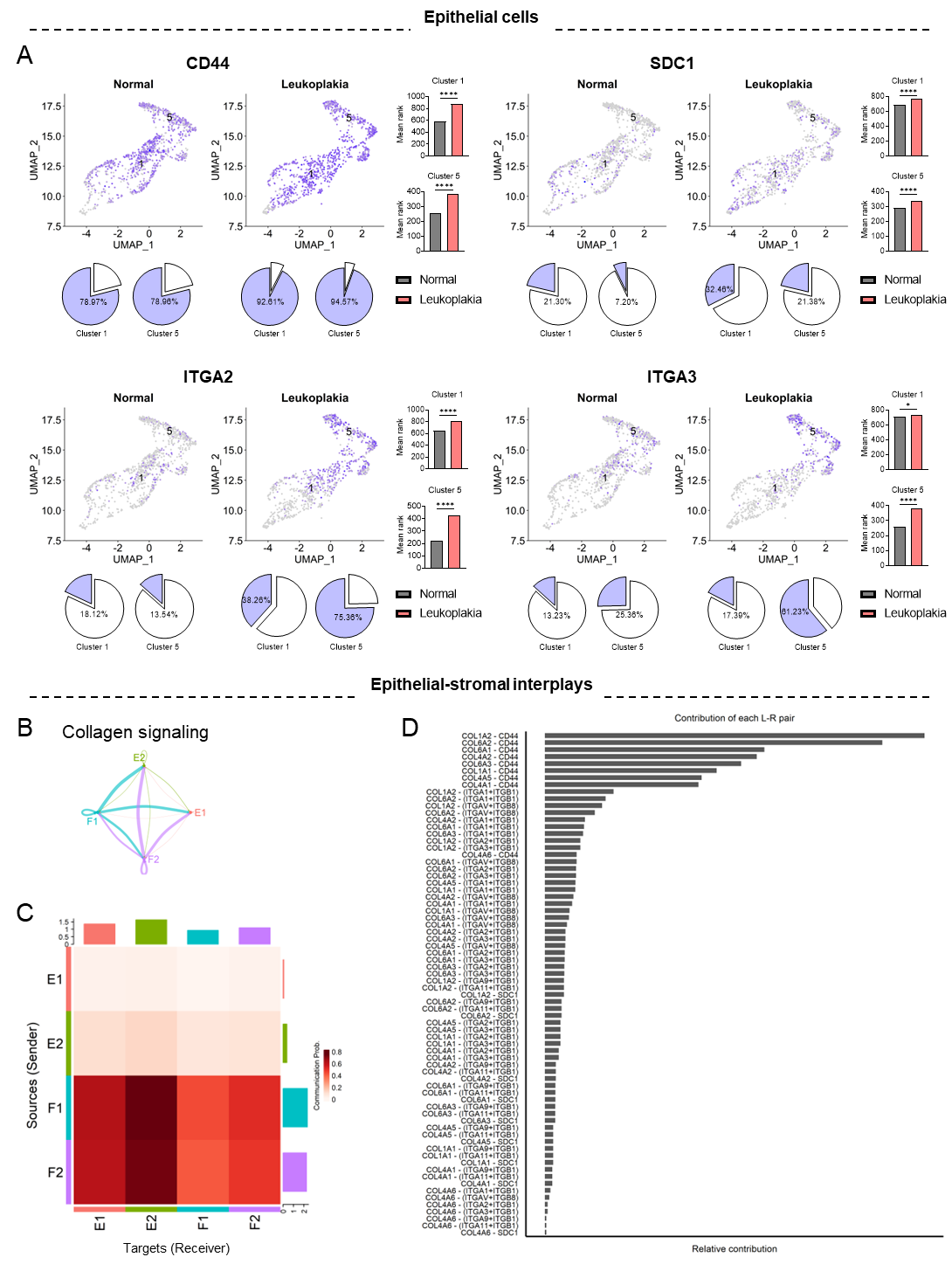


**Figure S18. Collagen signaling between fibroblasts and epithelial cells. (A)** The expression of collagen receptor genes, including *CD44*, *SDC1*, *ITGA2*, and *ITGA3*, is visualized for epithelial cell clusters (cluster 1 and 5) in the snRNA-seq dataset. The pie charts indicate the percentage of positive nuclei for each gene within the respective epithelial cell clusters. The bar plots indicate the mean rank$\pm$s.e.m. for gene expression. Statistical significance was assessed using the Mann-Whitney U test, with a *p*-value threshold of less than 0.05. **(B-D)** Cell-cell communication analysis reveals significant collagen signaling between epithelial cells and fibroblasts in the snRNA-seq dataset. **(B)** The circle plot illustrates the communication score between interacting cell clusters, with line thickness indicating the strength of communication. **(C)** The heatmap depicts the sender-receiver interaction matrix, where rows and columns represent sources and targets, respectively. The bar plots on the right and top represent the total outgoing and incoming interaction scores, respectively. **(D)** The relative contribution of ligand (L)-receptor (R) pairs involved in the communication is shown.
